# A proteomic‐based investigation of potential copper‐responsive biomarkers: Proteins, conceptual networks, and metabolic pathways featuring *Penicillium janthinellum* from a heavy metal‐polluted ecological niche

**DOI:** 10.1002/mbo3.485

**Published:** 2017-05-09

**Authors:** Xin Feng, Jian Xu, Yu Liang, Guo‐Li Chen, Xian‐Wei Fan, You‐Zhi Li

**Affiliations:** ^1^ State Key Laboratory for Conservation and Utilization of Subtropical Agro‐bioresources College of Life Science and Technology Guangxi University Nanning Guangxi China

**Keywords:** Cu tolerance, environment, fungus, pathways, protein network, proteome

## Abstract

Filamentous fungi‐copper (Cu) interactions are very important in the formation of natural ecosystems and the bioremediation of heavy metal pollution. However, important issues at the proteome level remain unclear. We compared six proteomes from Cu‐resistant wild‐type (WT) *Penicillium janthinellum* strain GXCR and a Cu‐sensitive mutant (EC‐6) under 0, 0.5, and 3 mmol/L Cu treatments using iTRAQ. A total of 495 known proteins were identified, and the following conclusions were drawn from the results: Cu tolerance depends on ATP generation and supply, which is relevant to glycolysis pathway activity; oxidative phosphorylation, the TCA cycle, gluconeogenesis, fatty acid synthesis, and metabolism are also affected by Cu; high Cu sensitivity is primarily due to an ATP energy deficit; among ATP generation pathways, Cu‐sensitive and Cu‐insensitive metabolic steps exist; gluconeogenesis pathway is crucial to the survival of fungi in Cu‐containing and sugar‐scarce environments; fungi change their proteomes via two routes (from ATP, ATP‐dependent RNA helicases (ADRHs), and ribosome biogenesis to proteasomes and from ATP, ADRHs to spliceosomes and/or stress‐adapted RNA degradosomes) to cope with changes in Cu concentrations; and unique routes exist through which fungi respond to high environmental Cu. Further, a general diagram of Cu‐responsive paths and a model theory of high Cu are proposed at the proteome level. Our work not only provides the potential protein biomarkers that indicate Cu pollution and targets metabolic steps for engineering Cu‐tolerant fungi during bioremediation but also presents clues for further insight into the heavy metal tolerance mechanisms of other eukaryotes.

## 1 Introduction

Ecology is the study of the properties that are required to survive in the environment (Winkelmann, 2007). Microbe–heavy metal (HM) interactions may alter the natural and man‐made ecosystems (Gadd, 2010; He, Yang, & Stoffella, 2005). In addition, Fungi‐HM interactions play roles in fundamental ecological processes and have considerable socio‐economic relevance (Gadd, 2007).

As eukaryotic microbes, filamentous fungi are quite different from eukaryotic yeasts and other eukaryotic organisms at least in morphology, and act as important players in the ecosystem (Fisher et al., 2012; Hoffmeister & Keller, 2007). Filamentous fungi living in special ecological niches have developed signature molecular metabolic pathways (Wisecaver & Rokas, 2015), for example, to enable survival in HMs‐rich environments (Keller, 2015). However, mechanisms of HM‐resistance are major issues in microbial ecology and environmental biotechnology (Mergeay et al., 2003) and seem to be species‐specific (Kuhn & Käufer, 2003), requiring deep insight from the global‐based perspective (García‐Sevillano et al., 2014).

Proteomics is an important tool for developing new potential and valuable protein biomarkers to indicate/monitor metal pollution in the environments (López‐Barea & Gómez‐Ariza, 2006) because protein type and copy number are determined in part by translational control (Ohno, Karagiannis, & Taniguchi, 2014). Proteomics has been branched into environmental research in the field of metallomics (López‐Barea & Gómez‐Ariza, 2006) and has been applied to large‐scale identification of HM‐responsive proteins of fungi such as yeasts (Irazusta, Estévez, Amoroso, & de Figueroa, 2012; López‐Barea & Gómez‐Ariza, 2006; Villegas, Amoroso, & de Figueroa, 2009), as well as of bacteria (Bar, Patil, Doshi, Kulkarni, & Gade, 2007), and plants (Dalcorso, Fasani, & Furini, 2013; Gill, Dogra, Kumar, Ahuja, & Sreenivasulu, 2012; Visioli & Marmiroli, 2013).

Copper (Cu) is a common trace HM element that is essential for living organisms (Kumpiene, Lagerkvist, & Maurice, 2008) but is toxic at high concentrations. Biochemical Cu toxicity is harmful to many enzymatic processes (van den Berghe & Klomp, 2010), primarily because it triggers the generation of reactive oxygen species (ROS) (Jomova & Valko, 2011), depending on species of organisms (Bao, Leung, Kwok, Zhang, & Lui, 2008; Lee et al., 2008). For instance, the 96‐hr median lethal concentration (LC50) values of Cu were 3.9 mg/L for adult *Tigriopus japonicas* (Lee et al., 2008), 78 μg/L for *Elasmopus rapax* and 970 μg/L for *Thalassiosira pseudonana* (Bao et al., 2008). Cu tolerance in organisms is associated with energy utilization (Cyert & Philpott, 2013; Dhar‐Chowdhury, Malester, Rajacic, & Coetzee, 2007; Visioli & Marmiroli, 2013; Voskoboinik, Mar, & Camakaris, 2003). Furthermore, regulation of Cu uptake and export is relevant to posttranslational modifications of proteins (van den Berghe & Klomp, 2010). To the best of our knowledge, only a few studies have focused on Cu‐responsive proteins in filamentous fungi such as *Aspergillus nidulans*s (Oddon, Diatloff, & Roberts, 2007) and *Botrytis cinerea*s (Cherrad et al., 2012). Nevertheless, most of these existing studies have only focused on the final result of ATP use during Cu resistance and have not considered the following during the resistance at the proteome level: of the steps in the ATP‐generating metabolic pathways; the mediator protein‐based networks in Cu signal transduction; and the changes in fungi proteomes and pathways in response to changes in Cu concentration.

We previously isolated a multi‐HMs resistant *Penicillium janthinellum* strain GXCR from HM‐polluted mining sludge in a mining area in Guangxi, China, which was resistant to Cu at a concentration of at least 200 mmol/L (Wei, Tang, Liang, Huang, & Li, 2006). Subsequently, through joint mutagenesis, we obtained the Cu mutant EC‐6, which exhibited decreased Cu resistance and failed to grow at 40 mmol/L Cu (Xu, Chen, Sun, Fan, & Li, 2015).

In this study, we conducted a proteomic analysis of the WT strain GXCR and its mutant EC‐6 under conditions of 0 (as control), 0.5 and 3 mmol/L Cu treatments using the high‐throughput isobaric tag for relative and absolute quantitation (iTRAQ) to look for Cu‐responsive protein features/biomarkers of filamentous fungi in harsh environments.

## 2 Materials and Methods

### 2.1 Fungal cultivation

The fungal strains used were the WT *P. janthinellum* strain GXCR with high resistance to Cu (Wei et al., 2006), and the mutant EC‐6, which exhibited decreased Cu resistance (Xu et al., 2015). The media used were liquid sugar‐less medium (SLM) which was composed of 1 g of yeast extract and 2 g of sodium gluconate in 1,000 ml of water; potato dextrose agar (PDA) composed of 1,000 ml of potato juice, 20 g glucose, and 20 g of agar; and potato dextrose juice without agar (PJ) consisted of 1,000 ml of potato juice and 20 g of glucose. The Cu salt used was CuSO_4_·5H_2_O. The mutant EC‐6 grew slowly in SLM containing more than 3 mmol/L Cu and failed to grow in SLM containing 5 mmol/L Cu when compared to the WT strain GXCR. The inocula used in this study were the full mature conidia collected near the center of the colonies grown for 10 day on PDA plates without added Cu.

To ensure synchronous growth of GXCR and EC‐6 to obtain the same number of mycelia in the presence of Cu, the conidia were therefore inoculated into 200 ml of SLM (with up to 1 × 10^6^ conidia/mL) without Cu as a control treatment or with 0.5 or 3 mmol/L Cu added as stress treatments. The inocula were then cultured for 3 day at 32°C in an incubator‐shaker at 200 rpm. After 3 days of cultivation, the culture contained mycelia that were in the initial stage of conidial sporulation. The mycelia were collected by filtration and then soaked for 2 min in 20 mmol/L Tris‐HCl (pH 8.5) containing 1 mmol/L EDTA and then washed twice with sterile ultrapure water to remove Cu ions on the surface of the mycelia. The mycelia were subsequently wrapped and gently squeezed using Whatman filter paper to remove the solution in the mycelial pellets and immediately frozen in liquid nitrogen and stored at −80°C.

### 2.2 Extraction of total proteins

For each particular treatment, equal weights of mycelia from three independent cultures were mixed and then pulverized in liquid nitrogen. The powdered mycelial materials were transferred into −20°C pre‐cooled acetone containing 10% trichloroacetic acid and fully mixed and stored at −20°C for 2 hr before centrifuging for 30 min at 20,000*g* and 4°C. The precipitate was suspended in pre‐cooled pure acetone, allowed to stand for 30 min at −20°C, and then centrifuged for 30 min at 20,000*g* and 4°C. This washing step with acetone was repeated several times until the precipitate became white. The acetone‐washed precipitate was transferred into lysis buffer containing 8 mol/L urea, 30 mmol/L HEPES, 1 mmol/L PMSF, 2 mmol/L EDTA, and 10 mmol/L DTT and then lysed for 5 min in an ultrasonic bath using a 2‐s pulse on and 3‐s pulse off sequence at 180 W. The lysate was centrifuged for 30 min at 20,000*g* and 4°C. The supernatant was collected, DTT was added to 10 mmol/L, and the sample was then heat‐treated for 1 hr in a 56°C water bath. Next, iodoacetamide was added to 55 mmol/L, and the sample was incubated for 1 hr in the dark before transferring it into a fourfold volume of pre‐cooled acetone solution and allowing it to stand at least for 3 hr at −20°C. Then the sample was centrifuged for 30 min at 20,000*g* and 4°C. The precipitate was dissolved using a 3‐min ultrasonic treatment in a solution composed of 50% triethylammonium bicarbonate (TEAB) and 0.1% sodium dodecyl sulfate (SDS). Then, the precipitate was centrifuged for 30 min at 20,000*g* and 4°C. The protein content in the supernatant was roughly analyzed through a conventional Bradford assay. The protein integrity was examined by SDS‐PAGE.

### 2.3 Protein digestion

For each of the specimens, the 100 μg protein extracts were adjusted to equal volumes using the TEAB solution containing 0.1% SDS. Then, 3.3 μg of the protease trypsin was added to the diluted protein and was pre‐digested for 24 hr in a 37°C water bath. Then, 1 μg of trypsin was added to the pre‐digestion solution and was digested for 12 hr in a 37°C water bath. The peptide fragment mix was freeze‐dried and then re‐dissolved for further use in ultrapure water‐TEAB solution at a ratio of 1(v):1(v).

### 2.4 Labeling of peptide fragments

An iTRAQ^®^ Reagent‐8Plex Multiplex Kit (Applied Biosystems) with isobaric tags was used to label peptides. The total mass of the isobaric tag was 305, which includes a reporter group with a mass from 113 to 121 (retains charge), not including 120, a balance group with a mass from 192 to 184 and amine‐ specific peptide reactive group. Each labeling reagent‐containing vial from the iTRAQ^®^ Reagent‐8Plex Multiplex Kit was fully mixed with 70 μl of isopropanol. The labeling reagent‐isopropanol mix was further mixed with 30 μl of the peptide fragment mix from each treatment, incubated for 2 hr at room temperature, and then freeze‐dried. For protein specimens from each treatment, three technical replicates were performed.

### 2.5 Analysis of strong cation exchange (SCX) high‐performance liquid chromatography (HPLC)

Solution A was composed of 25% ACN, 10 mmol/L KH_2_PO_4_, and 25% CAN. Solution B contained 2 mol/L KCl and 10 mmol/L KH_2_PO_4_. Both solutions A and B were adjusted to pH 3 with H_3_PO_4_, ultrasonicated for 5–10 min, and then filtered through an organic membrane with a 0.22‐μmol/L pore size before use. An Agilent SCX 1100 LC–G1312A instrument with two pumps (A and B) was used for chromatography. The chromatography column was a Luna SCX 100 Å column (250 mm × 4.6 mm; 100 Å; a 5‐μm particle diameter) (Phenomenex).

In brief, after opening the valves of the chromatography instrument, the pumps were filled with MilliQ water and then rinsed for 10 min with MilliQ water to exhaust and balance the pressure between the two pumps. Pumps A and B were filled with solutions A and B, respectively. Pump B was subsequently re‐balanced for 10 to 20 min at 1 mL/min with the solution A. After all the absorption peaks became flat, the protein specimen was loaded. For specimen loading, the freeze‐dried labeled peptide mix was diluted 10‐fold with solution A and then centrifuged for 10 min at 15,000*g* and 4°C. The diluted labeled peptide mix and solution B were loaded on a loop and then sequentially gradient‐eluted to separate and collect fractionized peptide fragments using the following procedures: 0.01 min starting with 0% solution B, 35 min with 0% solution B, 36 min with 5% solution B, 56 min with 30% solution B, 61 min with 50% solution B, 66 min with 50% solution B, 71 min with 100% solution B, and 81 min with 100% solution B and then stop in 81.01 min.

### 2.6 Peptide purification

The peptide fractions from SCX‐HPLC were desalted using reversed‐phase chromatography with a Strata‐X C18 column (100 mm ×75 mm; 300 Å; 5‐μm particle diameter) (Phenomenex). The column was activated with 1 ml of methanol at 2–3 drops/s and balanced with 5% ACN at 1 drop/s. An aliquot (1 ml) of the peptide solution was loaded on the column, eluted with 1 ml of 5% acetonitrile at 1 drop/s, and then eluted twice with 500 μl of 100% acetonitrile at a rate of 1 drop/s. The eluent was collected and then dried by centrifugation at 15,000*g* and 4°C. The acetonitrile‐free peptide pellet was dissolved in 0.1% formicacid.

### 2.7 Mass spectrometry detection

The Mass spectrometry detection (MSD) was conducted with peptide purification on a Q‐Exactive mass spectrometer (Thermo Fisher Scientific) coupled with an UltiMate3000 HPLC instrument (DIONEX, US) with a NanoLC C18 column (100 mm ×75 mm; 300 Å; 5‐μm particle diameter) (Jesen, Shanghai, China). Chromatographic separation was performed at 400 nl/min using the following parameters: positive ion mode, a first‐level scanning range of 350–6,000 Da (*m/z* 350–2,000) with a resolution of 70,000, an automatic second‐level scanning range with a resolution of 17,500, a capillary temperature of 20°C, a nano‐ESI ion source of 1,800 V, and high‐energy collisional dissociation fragmentation mode (NCE28; stepped NCE20%). The peptides were first eluted from the column with H_2_O‐0.1% FA solution A and then gradient‐eluted with 5% acetonitrile‐0.1% FA solution B at 0 min, with 5% solution B at 10 min, 30% solution B at 40 min, 60% solution B at 45 min, 80% solution B at 48 min, 80% solution B at 55 min, 5% solution B at 58 min, and 5% solution B at 65 min, before stopping at 65.01 min. The amount of protein loaded during each HLPC analysis was 1.5 μg.

### 2.8 Qualitative and quantitative protein analysis

The mass spectra (MS) were screened using Proteome Discoverer 1.3 software (Thermo) and the following run parameters: a parent ion mass range of 350‐6,000 Da, the number of 10 for the smallest peaks in the second‐level MS, and a signal to noise threshold of 1.5. The mass fragments were qualitatively analyzed by Mascot 2.3.0 according to the parameters described in Table S1, and then quantitatively analyzed by Proteome Discoverer 1.3 under the parameters described in Table S2. For each protein, at least 1 identified peptide was used for relative quantification of the protein level changes. The differentially expressed proteins (DPs) between specimens were defined as the difference in protein abundance at a threshold of >1.2 by using a *t* test with a significance of *p *<* *.05.

### 2.9 Functional annotation and pathway categorization of the proteins

The proteins were functionally annotated by Gene Ontology (GO) (http://www.geneontology.org/) and BLAST2GO (http://www.blast2go.com/b2ghome). The metabolic pathways of the proteins were designated by the KEGG Orthology‐Based Annotation System 2.0 (http://kobas.cbi.pku.edu.cn/help.do).

### 2.10 Quantitation of metabolites

Up to 10^4^ conidia/ml were inoculated into 100 ml of PJ containing 5 mmol/L Cu salt and cultured at 32°C in an incubator‐shaker at 200 rpm. The resulting mycelia were collected by filtration followed by washing with sterile deionized water (SDW). The mycelia were squeezed to remove water, washed in a solution containing 50 mmol/L Tris‐HCL and 10 mmol/L EDTA, and then washed again with SDW followed by squeezing to remove the water from the mycelia. These procedures were repeated three times. The mycelia were pulverized in liquid nitrogen. An aliquot (300 mg) of the powdered mycelial sample was fully mixed with a solution of 1,400 μl of methanol and 50 μl of internal standard containing 2 mg/ml adonitol (Sigma‐Aldrich, US) and then heat‐treated for 15 min in a 70°C water bathe. The mixture was cooled and then an equal volume of SWD was added before 750 μl of trichloromethane was added. This mixture was centrifuged for 10 min at 4°C and 3,200*g*. The supernatant was carefully transferred to a new Eppendorf tube and then vacuum‐dried for 16 hr at room temperature. The vacuum‐dried sample was re‐dissolved in 80 μl of a solution composed of 20 mg of methoxyamine hydrochloride (Sigma‐Aldrich, US) and 1 ml of pyridine and allowed to react for 90 min at 30°C. Then, a 40 μl aliquot of retention time standard mixture containing 0.029% (v/v) n‐dodecane, n‐pentadecane, n‐nonadecane, n‐docosane, n‐octacosane, n‐dotracontane, and n‐hexatriacontane dissolved in pyridine (Sigma‐Aldrich, US) as well as 80 μl of N‐methyl‐N‐(trimethylsilyl) trifluoroacetamide (Sigma‐Aldrich, US) was sequentially added and allowed to react for 30 min at 30°C. The reaction solution was used for the analysis of the metabolites using the gas chromatography (GC) and mass spectrometry (MSS).

GC was conducted on an Agilent 7890A GC System/5975C inert MSD mass spectrometer equipped with a 7683B autosampler, MSD ChemStation (HP/Agilent, US) and the National Institute of Standards and Technology (NIST) MSS database (HP/Agilent, US). The chromatographic column DB‐1701 (30 m × 250 μm × 0.25 μm, J&W) was used, which was operated for 5 min at a starting temperature of 100°C, heated to 280°C at 4°C/min and then maintained for 20 min at 280°C. A 1 μL aliquot of the sample solution was automatically injected into the column through the autosampler at a split ratio of 5:1, where the flow velocity of helium (the carrier gas) was 3 ml/min.

MSS was conducted using a quadrupole mass spectrometer (Agilent, US) with an electronic impact of 70 eV, an ion source temperature of 230°C,a transmission line temperature of 250°C, 2 scans/s, and a mass scan range of 50–600 m/z. The metabolites were then searched, recognized and identified according to m/z and retention time against the NIST database, with a matching degree of over 75%.

The raw MSS data were normalized, and a response ratio (RR) of each metabolite was calculated as its peak area divided by the standard peak area. The relative response value (RRR) for each metabolite was calculated as the RR divided by the sample mycelia weight.

Control treatments were conducted in parallel in mycelial culture conditions without the addition of Cu salt. Each treatment was independently analyzed using GC‐MSS with three independent culture batches. The level of each metabolite is represented as the mean of RRRs from three independent culture batches. Differential levels of metabolites were defined with fold changes observed in fungi under Cu stress compared with the relevant control under a significant difference level of *p < *.01 determined using Student's *t* test.

## 3 Results

### 3.1 Repertoire of proteins

**Figure 1:**
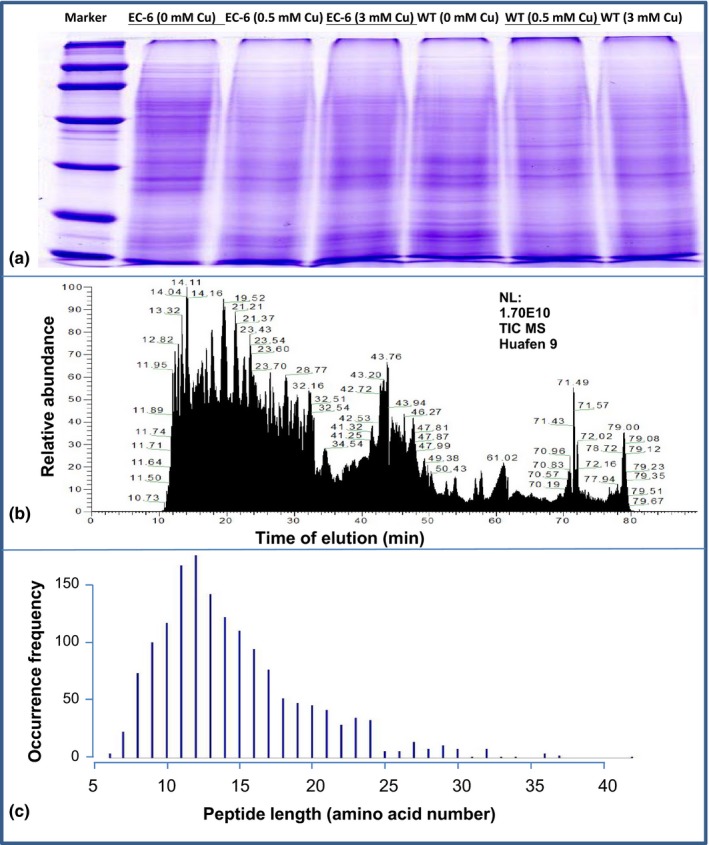
The proteins extracted from the strains under different treatments and analyzed with SDS‐PAGE (a), the schematic diagram of MSD (b), and length distribution of the detected peptides (c). EC‐6, mutant with decreased Cu resistance. mM, mmol/L; MSD, mass spectrometry detection; SDS‐PAGE, sodium dodecyl sulfate polyacrylamide gel electrophoresis; WT, wild‐type strain GXCR

The proteomes from a total of six specimens were prepared (Figure 1a) and then analyzed using iTRAQ. The total ion chromatogram from the peptide signals is showcased in Figure 1b. Further, 271,624 spectra were obtained, of which 6,425 were matched to the Swissprot_Fungin database. Of the matched spectra, 1,321 peptides were identified and the peptides with a high occurrence frequency (more than 50 times) were those comprising 8–17 amino acids (Figure 1c). Finally, a total of 495 proteins were determined with a false discovery rate of <1% (Table S3).

### 3.2 High‐abundance proteins

The majority (55.8%) of the identified proteins were related to basic metabolism. The high‐abundance proteins included heat shock proteins (Hsps) (4.2%), 60S ribosomal proteins(RPs)/ATP‐dependent RNA helicases (ADRHs) (3.4%), 40S RPs/eukaryotic translation initiation factors (3.2%), actin (2.6%), elongation factor 1 (2.2%), histone/26S protease regulatory subunit (2.0%), and proteasome/protein transport proteins (1.8%) (Table S4). In the protein repertoire there were catalase and catalase‐peroxidase (CP) (1.2%), superoxide dismutase (SOD) (1.0%), 14‐3‐3 proteins (0.4%), and spermidine synthase (SS) (0.2%) (Table S4).

### 3.3 DPs

**Figure 2:**
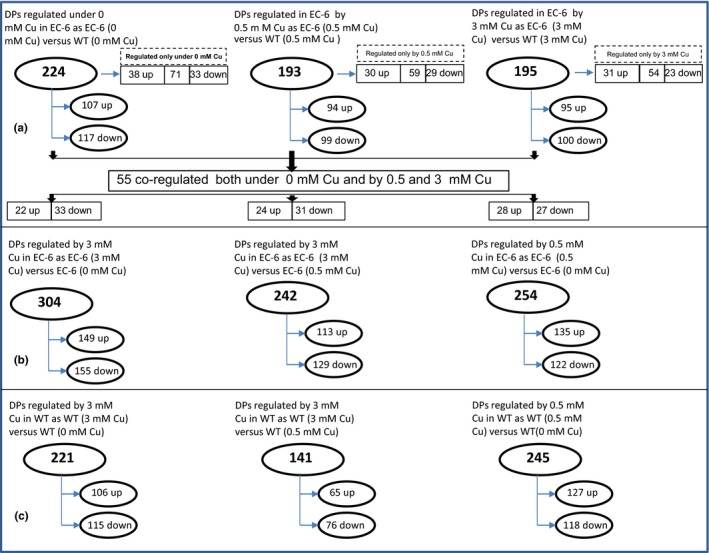
The DPs in EC‐6 compared with WT (a), in Cu‐treated EC‐6 (b), and in Cu‐treated WT (c). The MS were qualitatively analyzed using Mascot 2.3.0 software according to the given parameters. The DPs between the specimens were defined at a threshold of >1.2 and a test of *p *<.5. DPs, differentially expressed proteins; EC‐6, mutant with decreased Cu resistance; mM, mmol/L; MS, mass spectra; WT, wild‐type strain GXCR

When comparing the EC‐6 strain versus WT under the same conditions, there were 224 DPs in EC‐6 under the control conditions, and 193 and 195 DPs in EC‐6 under the 0.5 and 3 mmol/L Cu conditions (Figure 2a; Tables S5–S7), respectively. In addition, there were only 70, 59 and 54 DPs in EC‐6 only under the control, 0.5 mmol/L Cu and 3 mmol/L Cu treatments (Figure 2a; Table S8), respectively. The abundances of all 4 CPs and 3 Hsps decreased under the control conditions but increased under the 0.5 and 3 mmol/L Cu conditions (Table S9). The abundances of all three glyceraldehyde‐3‐phosphate dehydrogenases significantly decreased under the control and 3 mmol/L Cu treatments but increased under the 0.5 mmol/L Cu treatments (Table S9). The abundances of all three SODs significantly decreased under of the control, 0.5 mmol/L Cu and 3 mmol/L Cu treatments (Table S9). There were 55 DPs that were regulated in EC‐6 under all the treatments (Figure 2a; Table S10).

In comparing as EC‐6 versus EC‐6 (Figure 2b) or WT versus WT (Figure 2c) under the different treatments, many DPs were found in each strain, of which some showed a response either to a specific treatment or to all the treatments (Tables S11–S16).

### 3.4 DPs of interest

**Figure 3:**
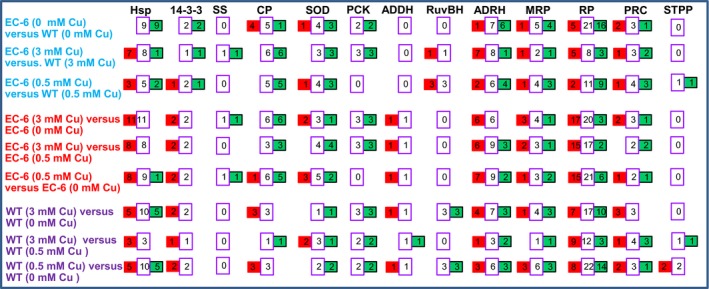
The DPs of interest in EC‐6 compared with WT, in Cu‐treated EC‐6, and in Cu‐treated WT. Red rectangles indicate the number of DPs with increased abundance. Green rectangles show the number of DPs with decreased abundance. The middle lilac rectangles between the red and green rectangles indicate the total number of DPs. ADDH, ATP‐dependent DNA helicase II; ADRH, ATP‐dependent RNA helicase; CP, catalase‐peroxidase; DPs, differentially expressed proteins; EC‐6, mutant with decreased Cu resistance; Hsp, heat shock protein; 14‐3‐3, 14‐3‐3 protein; mM, mmol/L; MRP, mitochondria‐related protein (cytochrome c oxidase, cytochrome c peroxidase, cytochrome c 1, and mitochondrial presequence protease); PCK, phosphoenolpyruvate carboxykinase; PRC, proteasome component; RP, ribosomal protein; RuvBH, ATP‐dependent RuvB helicase; SOD, superoxide dismutase; SS, spermidine synthase; STPP, serine/threonine‐protein phosphatase; WT, wild‐type strain GXCR

Special attention was given to the following DPs: Hsps (Feder & Hofmann, 1999); 14‐3‐3 protein homologs (14‐3‐3) (Roberts, Salinas, & Collinge, 2002); CPs and SODs (Li, Harvey, & McNeil, 2009), SS (Pegg, 2014); phosphoenolpyruvate carboxykinase (PCK) (Jardón, Gancedo, & Flores, 2008); serine/threonine‐protein phosphatase; organelles‐related proteins, namely RPs and proteasome components (PRCs), and the mitochondria‐related proteins (MRP) such as cytochrome c oxidase (COX) (Puig & Thiele, 2002), cytochrome c peroxidase, cytochrome c 1, and mitochondrial presequence protease; and three DNA/RNA unwinding‐related proteins, namely ATP‐dependent DNA helicase II (ADDH) and ADRHs (Jankowsky, 2011; Pyle, 2008), ATP‐dependent RuvB helicases (RuvBHs) (Ohdate et al., 2003), and pyruvate carboxylase (PC). Overall, under Cu stress, the abundances of both Hsp and CP significantly increased; the abundance of SS, SODs and PCK decreased; and the abundances of ADDHs, RuvBHs, ADRHs, and MRPs significantly increased. PC abundance was higher in EC‐6 than in WT under the control conditions and lower in EC‐6 than in WT under the Cu treatments (Figure 3; Tables S5–S7).

### 3.5 Mapping of DPs onto metabolic pathways

**Figure 4:**
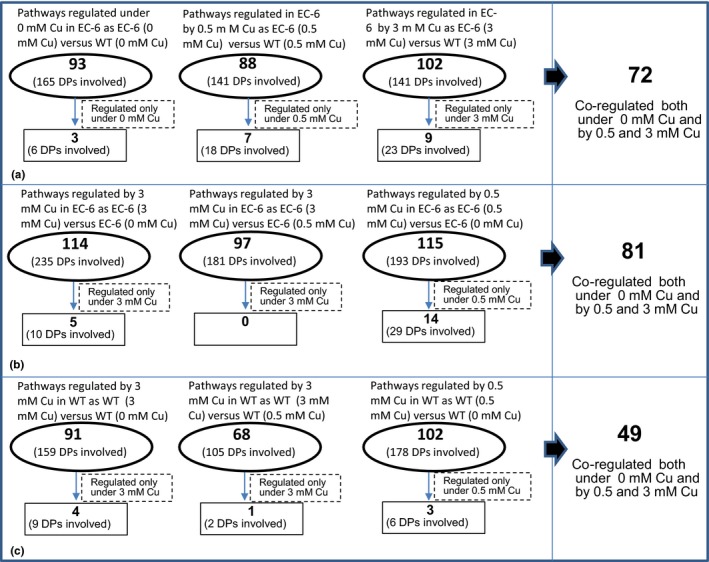
The summary of the total metabolic pathways differentially regulated in EC‐6 compared with WT (a), in Cu‐treated EC‐6 (b), and in Cu‐treated WT (c). The metabolic pathways of the proteins were designated by KEGG Orthology‐Based Annotation System 2.0 software (http://kobas.cbi.pku.edu.cn/help.do). DPs, differentially expressed proteins; EC‐6, mutant with decreased Cu resistance; mM, mmol/L; WT, wild‐type strain GXCR

When comparing as EC‐6 versus WT under control conditions, a total of 93 pathways were affected in EC‐6, involving 165 DPs (67.3%) (Figure 4a; Table S17).

Comparing EC‐6 versus WT under 0.5 mmol/L Cu stress, 88 pathways were significantly affected in EC‐6, covering 141 (73%) DPs (Figure 4a; Table S18).

In EC‐6 versus WT under 3 mmol/L Cu, 102 pathways were dysregulated in EC‐6 (Figure 4a; Table S19), covering 141 (72.3%) DPs. Some pathways which were co‐regulated by 0, 0.5 and 3 mmol/L Cu (Table S20), and others were regulated only by a specific treatment in EC‐6 when comparing EC‐6 versus WT (Table S21).

Comparing EC‐6 versus EC‐6 or WT versus WT, some condition‐specific pathways were identified in EC‐6 or WT (Figure 4b and c; Tables S22–S31). Notably, no dysregulated pathways were found in EC‐6 under 3 mmol/L Cu compared with EC‐6 under 0.5 mmol/L Cu treatment (Figure 4b; Table S23).

### 3.6 Glycolysis and gluconeogenesis pathways

**Figure 5:**
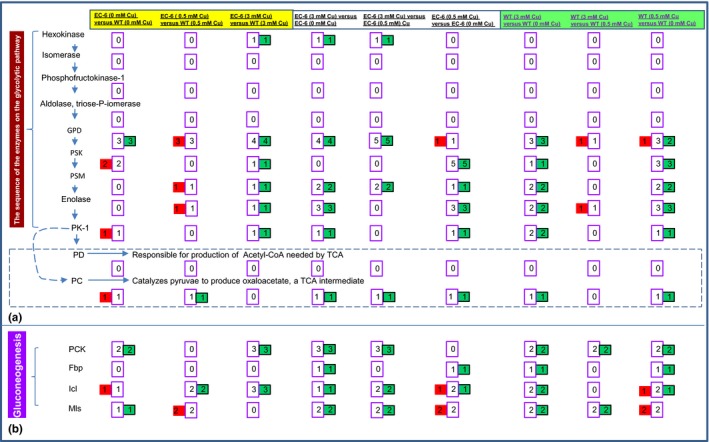
The affected enzymes in the glycolytic pathway (a), and gluconeogenesis pathway (b). The enzymes and their order in the pathways (Jardón et al., 2008; Kern et al., 2007; Rui, 2014; Yin et al., 2015). Red rectangles indicate the number of DPs with increased abundance. Green rectangles show the number of DPs with decreased abundance. The middle purple rectangles between the red and green rectangles indicate the total number of DPs. DPs, differentially expressed proteins. EC‐6, mutant with decreased Cu resistance; Fbp, fructose‐1,6‐bisphosphatase; GPD, glyceraldehyde‐3‐phosphate dehydrogenase; Icl, isocitrate lyase; Mls, malate synthase; mM, mmol/L; OP, oxidative phosphorylation; PC, pyruvate carboxylase; PCK, phosphoenolpyruvate carboxykinase; PD, pyruvate dehydrogenase; PK, pyruvate kinase; PSK, phosphoglycerate kinase; PSM, phosphoglycerate mutase; WT, wild‐type strain GXCR

ATP production involves glycolysis, oxidative phosphorylation (OP), the citrate cycle (i.e., the TCA cycle), and fatty acid oxidation (Akram, 2014; Dhar‐Chowdhury et al., 2007). Glycolysis is coupled with both gluconeogenesis and the TCA cycle (Jardón et al., 2008) and was greatly affected in the strains under Cu treatment (Tables S17‐S19, S22‐S24, and S27‐S29). Notably, Cu stress primarily attacked the later catalytic steps of glycolysis (Figure 5a; Figures S1–S7).

As for mediators between glycolysis and the TCA cycle (Porporato, Dhup, Dadhich, Copetti, & Sonveaux, 2011), pyruvate dehydrogenase (PD) was not affected in either of the two strains under the control or Cu treatments or in EC‐6 compared with WT. The abundance of PC significantly increased in EC‐6 compared with WT under the control conditions but decreased in EC‐6 compared with WT under the 0.5 mmol/L Cu treatment. However, the abundance of PC significantly decreased in each of the two strains under the Cu treatment when EC‐6 (3 or 0.5 mmol/L Cu) and EC‐6 (Control) or WT (3 or 0.5 mmol/L Cu) and WT (Control) were compared (Figure 5a).

The abundances of the enzyme proteins in gluconeogenesis (Jardón et al., 2008) were significantly reduced in both EC‐6 and WT, especially under the 3 mmol/L Cu treatment (Figure 5b).

### 3.7 The TCA pathway

**Figure 6:**
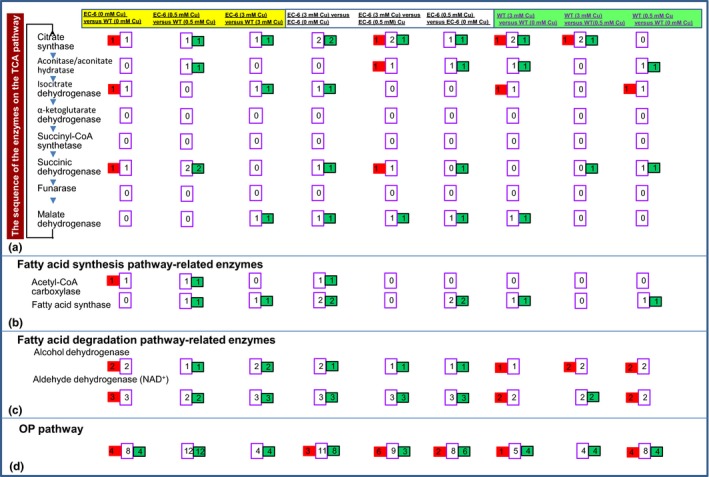
The affected enzymes in the TCA pathways (a), fatty acid synthesis‐related pathway (b), fatty acid degradation‐related pathway (c), and OP pathway (d). The enzymes and their order in the pathways (Akram, 2014; Bastin, 2014; Dhar‐Chowdhury et al., 2007; Jardón et al., 2008; Kern et al., 2007; Rui, 2014; Yin et al., 2015). Red rectangles indicate the number of DPs with increased abundance. Green rectangles show the number of DPs with decreased abundance. The middle lilac rectangles between the red and green rectangles indicate the total number of DPs. DPs, differentially expressed proteins; EC‐6, mutant with decreased Cu resistance; mM, mmol/L; OP, oxidative phosphorylation; TCA cycle, tricarboxylic acid cycle (citric acid cycle); WT, wild‐type strain GXCR

When comparing EC‐6 (0 mmol/L Cu) with WT (0 mmol/L Cu), the activities of the TCA pathway seemed to be enhanced in EC‐6 because the abundances of the citrate synthase, isocitrate dehydrogenase, and succinic dehydrogenase were increased (Figure 6a; Figure S8). Comparing EC‐6 (0.5 mmol/L Cu) with WT (0.5 mmol/L Cu), the abundances of citrate synthase, aconitase/aconitate hydratase, and succinic dehydrogenase significantly decreased in EC‐6 (Figure 6a; Figure S9). Comparing EC‐6 (3 mmol/L Cu) and WT (3 mmol/L Cu), the abundances of citrate synthase, isocitrate dehydrogenase, and malate dehydrogenase significantly decreased in EC‐6 (Figure 6a; Figure S10). Simultaneously, the abundances of other related DPs significantly decreased in EC‐6 and WT in the Cu treatments when comparing either EC‐6 (Cu stress) and EC‐6 (Control) or WT (Cu stress) and WT (Control) (Figure 6a; Figures S11–S14).

### 3.8 Fatty acid synthesis and metabolic pathways

Fatty acids supply ATP via β oxidation (Bastin, 2014). Among the enzymes associated with fatty acid synthesis (Rui, 2014), Cu stress in both EC‐6 and WT only led to decreased abundances of acetyl‐CoA carboxylase and fatty acid synthase (Figure 6b; Figures S15–S21).

In the fatty acid degradation pathway, Cu stress only affected alcohol dehydrogenase and aldehyde dehydrogenase (NAD^+^) (Figures S15–S21). Comparing EC‐6 (0 mmol/L Cu) versus WT (0 mmol/L Cu), these two enzymes showed the increased abundances in EC‐6 (Figure 6c; Figure S15). In comparing EC‐6 (0.5 mmol/L Cu) versus WT (0.5 mmol/L Cu) and EC‐6 (3 mmol/L Cu) versus WT (3 mmol/L Cu), the abundances of the enzymes significantly decreased in EC‐6 (Figure 6c; Figures S16 and S17). Notably, when comparing EC‐6 (0.5 mmol/L Cu) versus EC‐6 (0 mmol/L Cu) and EC‐6 (3 mmol/L Cu) versus EC‐6 (0 mmol/L Cu), the abundances of the enzymes also significantly decreased in EC‐6 (Figures S18 and S19). When comparing WT (0.5 mmol/L Cu) versus WT (0 mmol/L Cu) and WT (3 mmol/L Cu) versus WT (0 mmol/L Cu), increased abundances were observed in WT (Figures S20 and S21).

### 3.9 The OP pathway

DPs mapped onto the OP pathway included ATP synthases, V‐type proton ATPases, inorganic pyrophosphatases, COX, cytochrome c, succinate dehydrogenases, and NADH‐ubiquinone oxidoreductases (Tables S17‐S19, S22‐24, and S27‐S29). Most of these proteins showed decreased abundance in EC‐6 or WT under Cu treatment. However, all the DPs involved in the pathway showed decreased abundance in EC‐6 under Cu when comparing EC‐6 versus WT (Figure 6d).

**Figure 7:**
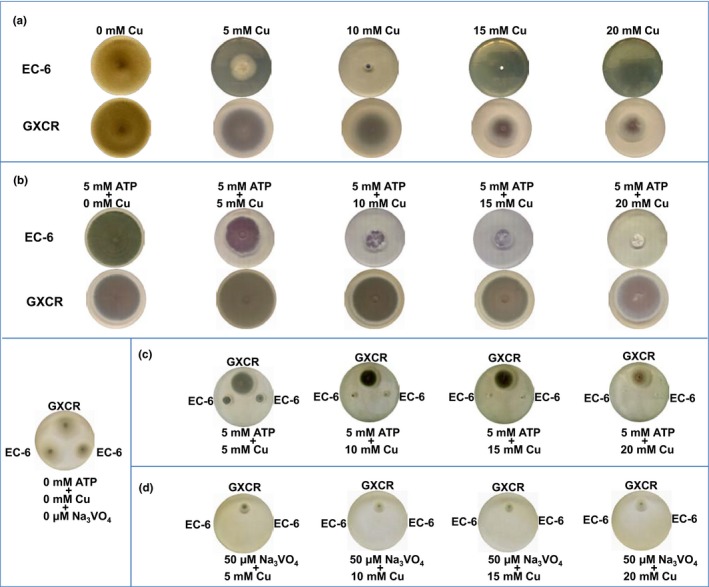
The phenotypes of WT and EC‐6 grown on PDA plates for 10 day (a and b) and for 5 day (c and d) at 32°C under Cu, ATP, the ATPase inhibitor Na_3_ VO_4_ or a combination. Pictures are shown of colonies formed from cultures started with equal amounts of mycelia and grown on PDA plates for 10 day at 32°C. The results represent at least three different batches of experiments. EC‐6, mutant with decreased Cu resistance; mM, mmol/L; WT, wild‐type strain GXCR

Both mutant EC‐6 and WT strain GXCR showed increased Cu tolerance on Cu‐containing PDA plates supplemented with 5 mmol/L ATP (Figure 7b) when compared to the respective growth in the presence of Cu alone (Figure 7a), but EC‐6 still had significantly lower growth than GXCR (Figure 7c). When co‐cultured on the same PDA plate, EC‐6 failed to grow under conditions with both Cu and the ATPase inhibitor Na_3_VO_4_ present (Aladdin, Shanghai, China) (Figure 7d).

### 3.10 Signaling pathways

**Figure 8:**
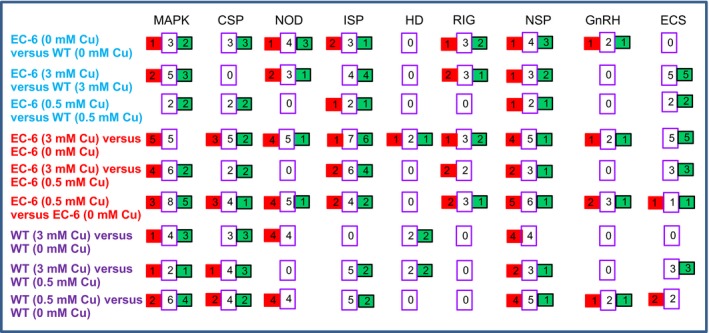
Cu‐responsive responsive signaling pathways. Red rectangles indicate the number of DPs with increased abundance. Green rectangles show the number of DPs with decreased abundance. The middle lilac rectangles between the red and green rectangles indicate the total number of DPs. CSP, calcium signaling pathway; DPs, differentially expressed proteins; EC‐6, mutant with decreased Cu resistance; ECS, epithelial cell signaling in *H. pylori* infection; GnRH, GnRH signaling pathway; GXCR **,** wild‐type strain; HD, Hedgehog signaling pathway; ISP, insulin signaling pathway; MAPK, MAPK signaling pathway; mM, mmol/L; NOD, NOD‐like receptor signaling pathway; NSP, neurotrophin signaling pathway; RIG, RIG‐I‐like receptor signaling pathway; WT, wild‐type strain GXCR

The Cu stress‐responding signaling pathways included the calcium signaling pathway (CSP), GnRH signaling pathway (GnRH), Hedgehog signaling pathway (HD), insulin signaling pathway (ISP), MAPK signaling pathway (MAPK), NOD‐like receptor signaling pathway (NOD), neurotrophin signaling pathway (NSP), Epithelial cell signaling in *Helicobacter pylori* infection (ECS), and the RIG‐I‐like receptor signaling pathway (RIG). Of these pathways, some responded only to a certain concentration of Cu (Figure 8; Tables S17‐S19, S22‐S24, and S27‐31), depending on the strain.

### 3.11 Changes in the levels of metabolites in glycolysis and the TCA pathway under Cu stress

Enzymes catalyze the formation of the metabolites. In view of the activity changes in the above protein enzymes in glycolysis and the TCA pathway enzymes, we assayed the metabolite levels in the WT strain GXCR cultured with 5 mmol/L Cu over a period of time. Consequently, in the WT strain under Cu, levels of glucose, glycerol, and D‐fructose significantly decreased, and the level of glycerol‐3‐phosphate (G3P) were significantly increased (Table 1). Citrate was not affected 24 hr after the stress but sharply increased 48 hr after the stress (Table 1). Succinic acid, fumaric acid, and malic acid were all decreased within 48 hr of the stress (Table 1).

**Table 1:** Changes in amount of metabolites in glycolysis and TCA pathways in WT strain GXCR under Cu stress

Metabolite	After 5 mmol/L Cu stress					
	24 hr (Fold change)	*P* at *t* test	48 hr (Fold change)	*P* at *t* test	72 hr (Fold change)	*P* at *t* test
Glucose	0.317 ± 0.012	2.074E‐06	0.388 ± 0.006	5.301E‐07	0.298 ± 0.008	2.863E‐05
D‐fructose	0.051 ± 0.010	0.000	0.297 ± 0.003	0.000	0.582 ± 0.021	0.000
Glycerol	0.231 ± 0.006	7.926E‐06	0.348 ± 0.038	0.00017	1.320 ± 0.139	0.053
Glycerol‐3‐phosphate	707.319 ± 99.002	0.000	17.643 ± 0.536	9.145E‐05	1237.336 ± 103.681	3.246E‐05
Citrate	0.00	0.013	64.149 ± 4.300	1.429E‐05	0.00	0.158
Succinic acid	0.128 ± 0.007	2.479E‐06	0.148 ± 0.014	0.000	0.519 ± 0.052	0.013
(E)‐2‐Butenedioic acid/Fumaric acid	0.057 ± 0.003	2.479E‐06	0.256 ± 0.025	0.000	0.558 ± 0.014	0.013
Malic acid	0.208 ± 0.005	3.672E‐06	0.304 ± 0.013	0.002	0.568 ± 0.006	0.013

Mycelial culture started from conidia and grown in PJ was used for analysis of metabolites by GC‐MSS. The figures show the fold changes in metabolic levels in Cu‐stressed WT strain GXCR as a comparison of stress (5 mmol/L Cu) vs. concurrent control (0 mmol/L Cu) conditions. Each treatment was independently analyzed using GC‐MSS with three independent batches of culture. Each datum is the mean of RRRs from three independent batches of culture. Increased levels of metabolites are marked in red. Decreased levels of metabolites are highlighted in green. The values following “±” are standard deviations of three batches of repeats. GC‐MSS, gas chromatography and mass spectrometry; MS, mass spectra; PJ, medium consisting of potato infusion and glucose. RR, response ratio from analysis of the MS. RRRs, relative response values, calculated as RR divided by the sample mycelia weight; WT, wild‐type.

## 4 Discussion

iTRAQ is conceptually elegant and widely employed in discovery‐based proteomics and allows for simultaneous protein identification and (relative) quantification obtained at a relatively low mass reporter ion intensity (Evans et al., 2012). In this study, the number of identified protein was relatively small likely because the iTRAQ technique itself has a low mass reporter ion intensity, which reduces the number of quantifiable and identifiable peptides, especially for low‐abundance proteins (Chen et al., 2012). On the other hand, hydrophobic or low abundance plasma membrane proteins are often difficult to extract, and their abundance is beyond the detection limits of standard proteomic techniques (Komatsu, Konishi, & Hashimoto, 2007). Additionally, although the 8.0 mol/L urea‐trypsin system used in this study is very popular for protein extraction and proteolytic digestion in iTRAQ, this digestion system possibly decreases the number of identified proteins because of the effect of proteolytic digestion on MS identification and because of interference with stable isotope‐labeling in iTRAQ (Kollipara & Zahedi, 2013). Certainly, some proteins are likely secreted, which occurs in *B. cinerea*s under heavy metal treatment (Cherrad et al., 2012), resulting in a lower number of intracellular proteins.

Among the proteins framed in the Cu tolerance model of yeasts (Cyert & Philpott, 2013; Puig & Thiele, 2002), some well‐known heavy metal ion transporter proteins and metallothionines were not found in our protein repertoire. A similar result was obtained in another proteomic study of the yeast strain *Rhodotorula mucilaginosa* RCL‐11 under Cu stress (Irazusta et al., 2012). However, a large number of the mRNAs coding various cation transporter proteins was found in the large‐scale transcriptome sequencing of GXCR and EC‐6 under Cu stress (Xu et al., 2015). Proteins have a longer lifetime than mRNA (Ohno et al., 2014). Therefore, failure to identify these proteins likely resulted from their low abundance and occurred because membrane proteins are usually underrepresented.

Some proteins, such as Hsps (Feder & Hofmann, 1999), 14‐3‐3 proteins (Roberts et al., 2002), CPs and SODs (Li et al., 2009), and SS (Pegg, 2014) have tolerance to abiotic stress. Our data suggests that these proteins are also associated with Cu tolerance in fungi owing to their active changes in abundance in GXCR and EC‐6 under Cu stress (Figure 3).

Under physiological conditions, free Cu very rarely exists inside cells, with an upper limit that is much lower than a single atom per cell (Jomova & Valko, 2011). Unlike other heavy metals, Cu seems more likely to induce the generation of ROS (Jomova & Valko, 2011), suggesting that oxidative stress caused by ROS is one of the earliest physiological stress response events that occur in the cell under Cu. The ECS, MAPK, NOD and NSP signaling pathways are directed to DNA replication, and CSP, ISP and RIG are associated with the mitochondria, although CSP and MAPK exhibit cross‐talk (Figure 8; Tables S17–S22, and S25–S27). ADDHs and RuvBHs are ATP‐dependent and regulate DNA unwinding and reconfiguration of nucleosomal structures (Jónsson et al., 2001; Ohdate et al., 2003; Pyle, 2008), facilitating transcription initiation. ADRHs are also ATP‐dependent RNA helicases and function in ribosome biogenesis, and pre‐mRNA splicing and translation in the nucleus and mitochondria (Jankowsky, 2011). In addition, ADRHs contribute to the formation of RNA degradosomes and stabilize RNA secondary structure in prokaryotes under abiotic stress (Owttrim, 2013). These ADDHs and ADRHs were actively responsive to Cu stress in both WT and EC‐6 (Figure 3). Changes in RNA metabolic pathways can contribute to the cellular tolerance of butanol stress (Owttrim, 2013). Therefore, Cu stress signals were likely transmitted via the above‐mentioned signaling pathways to ADDHs, RuvBHs, and ADRHs in the nucleus and to ADRHs in the mitochondria, consequently affecting the transcription and translation in both organelles. ADRHs function in the remodeling of spliceosomes, which not only function in pre‐mRNA splicing (Jankowsky, 2011; Jarmoskaite & Russell, 2014) but also act as a major component of stress‐adapted RNA degradosomes which have been speculated to form in order to degrade abnormal RNAs in bacteria under stress (Owttrim, 2013; Pyle, 2008; Steimer & Klostermeier, 2012).

Some DPs (Table S8) and pathways (Tables S25 and S31) showed responses specific to certain Cu concentrations. Together, these results indicate that different protein‐based mechanisms are used by fungi to cope with low and high Cu.

Many metal ion transporters or hyperaccumulation processes are ATP‐driven or associated with energy metabolism (Cyert & Philpott, 2013; Dhar‐Chowdhury et al., 2007; Visioli & Marmiroli, 2013; Voskoboinik et al., 2003). When ATP production does not keep up with the demands, energy‐utilizing synthetic pathways are inhibited. Among the four ATP‐producing pathways, OP is committed to occur in electron transport chain complexes on the inner mitochondrial membrane to produce ATP (Akram, 2014; Dhar‐Chowdhury et al., 2007). Glucose‐6‐P in glycolysis is linked to the synthesis of trehalose (Jardón et al., 2008). Trehalose is a stress‐tolerance‐related metabolite (Elbein, Pan, Pastuszak, & Carroll, 2003). Acetyl‐CoA carboxylase, a link between glycolysis and the TCA pathway, is a precursor that initiates fatty acid biosynthesis through acetyl‐CoA carboxylase, which is stimulated by citrate (Akram, 2014). Glycolysis (Figure 5a), the TCA (Figure 6a) and OP (Figure 6d) were all significantly impaired especially in mutant EC‐6 under high (3 mmol/L) Cu (Figures 5 and 6), coinciding with the differential expression of a large number of ATP production‐related genes (Xu et al., 2015). Additionally, Cu tolerance in both the mutant EC‐6 and WT strain GXCR was significantly increased under exogenous ATP (Figure 7a and b). Although the WT strain GXCR still grew, EC‐6 failed to grow in the presence of both Cu and the ATPase inhibitor Na_3_VO_4_ (Figure 7d), which suggested that ATP energy was indeed relatively low in EC‐6. Overall, these results strongly indicate that Cu stress can lead to an ATP energy deficit in the cell and ATP energy is indispensable to Cu tolerance. Notably, some metabolic steps in the affected pathways were easily assaulted by Cu but other steps were not (Figures 5 and 6).

Typically, the gluconeogenesis is employed by microorganisms to produce available sugars such as glucose, from non‐sugar carbon sources or during carbon starvation to successfully colonize the sugar‐scarce niches/environments (Brock, 2009; Jardón et al., 2008). This pathway is accomplished in part by the enzyme PCK with oxaloacetate to bypass the physiologically irreversible pyruvate kinase step in glycolysis pathway (Brock, 2009; Jardón et al., 2008). Oxaloacetate is synthesized by the enzyme isocitrate lyase in the glyoxylate cycle (Brock, 2009). An increased amount of an available carbon source (glucose) can alleviate Cu toxicity toward the filamentous fungi (Gadd, Ramsay, Crawford, & Ritz, 2001). GXCR was isolated from a sugar source‐deficient and HM‐containing environment (Wei et al., 2006). The proteomics presented in this study were also conducted with a mycelial culture grown in a low‐sugar liquid medium. Most of the four enzymes in the gluconeogenesis pathway showed a significant decrease in abundance under Cu (Figure 5b). Therefore, the gluconeogenesis is crucial to the survival of the fungi in Cu‐containing and sugar‐scarce niches/environments.

**Figure 9:**
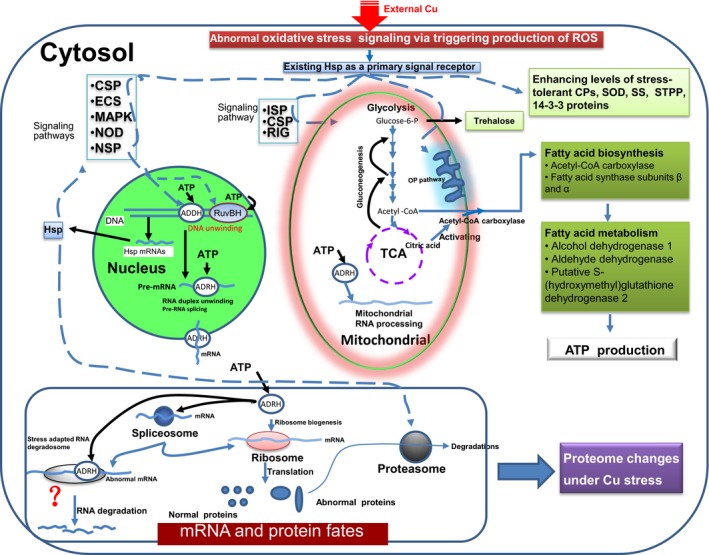
A general diagram of Cu‐responsive paths in the cells of filamentous fungi at the proteome level. This diagram depicts the paths and components that respond to Cu stress and indicates how the fungi change their proteomes to address changes in Cu concentration. The details have been described in the Discussion. The broken lines with arrows suggest the Cu stress signaling paths and involved components. ADDH, ATP‐dependent DNA helicase II; ADRH, ATP‐dependent RNA helicase; CP, catalase‐peroxidase; CSP, calcium signaling pathway; DPs, differentially expressed proteins; ECS, epithelial cell signaling in *H. pylori* infection; GnRH, GnRH signaling pathway; HD, Hedgehog signaling pathway; Hsp, heat shock protein; 14‐3‐3, 14‐3‐3 proteins; ISP, insulin signaling pathway; MAPK, MAPK signaling pathway; NOD, NOD‐like receptor signaling pathway; NSP, neurotrophin signaling pathway; OP, oxidative phosphorylation; RIG, RIG‐I‐like receptor signaling pathway; ROS, reactive oxygen species; RuvBH, ATP‐dependent RuvB helicase; SOD, superoxide dismutase; SS, spermidine synthase; STPP, serine/threonine‐protein phosphatase; TCA, citrate cycle

Hsps are extremely sensitive to even minor assaults (Gupta, Sharma, Mishra, Mishra, & Chowdhuri, 2010). Some Cu‐regulated Hsps were mapped on the signaling pathways affected by Cu (Tables S5‐S8, S10‐S19, and S22‐31), suggesting that changes in the abundances of Hsps can be used as early warning signs under Cu stress. Taking all the above discussions together, the general responses of the filamentous fungi to Cu and the processes associated with the changes in fungal protein levels in response to Cu concentrations were sketched out and are shown in Figure 9.

**Figure 10:**
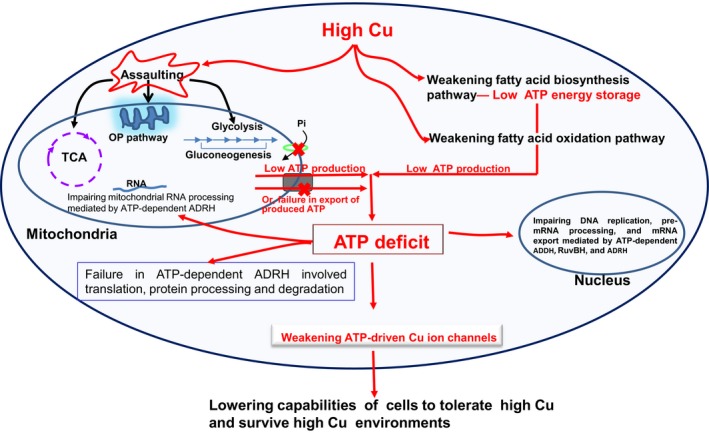
The diagram of high Cu sensitivity theory in filamentous fungi at the proteome level. This diagram emphasizes and explains a theory of high Cu sensitivity due to Cu stress‐induced ATP energy defects and indicates the key paths impaired by the presence of high levels of Cu and the key ATP‐dependent protein players ADDH, ADRH, and RuvBH. The details have been described in the Discussion. The black square box represents the voltage‐dependent anion channel; and the light green ovals indicate phosphate transporters. DPs, differentially expressed proteins; ADDH, ATP‐dependent DNA helicase II; ADRH, ATP‐dependent RNA helicase; OP, oxidative phosphorylation; Pi, inorganic phosphate; RuvBH, ATP‐dependent RuvB helicases; TCA cycle, tricarboxylic acid cycle (citric acid cycle)

Mitochondria are the first responders in the event of an imbalanced Cu homeostasis **(**Zischka & Lichtmannegger, 2014 **)** and act as the main places of Cu‐dependent production of cellular ATP energy (Kaniak‐Golik & Skoneczna, 2015; Nevitt, Ohrvik, & Thiele, 2012). Cu also acts as a structural component of some mitochondrial membrane proteins (Puig & Thiele, 2002). High Cu causes deformity of the mitochondrial cristae (Roberts, Robinson, & Yang, 2008), likely via peroxidation (Zischka & Lichtmannegger, 2014), and therefore likely leads to failure in the export of produced ATP out of the mitochondria and blocked import of inorganic phosphate (Pi) into mitochondria through phosphate transporters located in the mitochondrial inner membrane (Lemasters & Holmuhamedov, 2006). Pi is necessary for ATP production in mitochondria (Lemasters & Holmuhamedov, 2006). Therefore, we further proposed a conceptual model of high Cu sensitivity due to Cu stress‐induced ATP energy defects as shown in Figure 10.

Decreased levels of intermediate, not pathway‐initiating, metabolites in ATP‐producing pathways under Cu stress (Table 1) are likely to address the ATP need in ATP‐driven Cu resistance by accelerating pathway metabolism. Increased levels of intermediate metabolites (Table 1) indicate that step‐onward conversion of the metabolites through enzyme catalysis is affected. Decreased levels of glucose and D‐fructose (Table 1) indicate that the steps from glucose to fructose in glysolysis were not affected in WT strain GXCR under Cu. Glyceraldehyde‐3‐phosphate dehydrogenase (GPD) is responsible for conversion of G3P to glyceraldehyde‐3‐phosphate, and the latter is synthesized from glycerol (Kern, Tilley, Hunter, Legisa, & Glieder, 2007; Yin et al., 2015). The decreased GPD is bound to lead to high accumulation of G3P, consistent with the expected increase in G3P and decrease in glycerol determined with an assay in this strain of the GXCR (Table 1). The decreased succinic acid and fumaric acid under 5 mmol/L Cu stress (Table 1) indirectly showed that the succinate dehydrogenase‐catalyzed and fumarase‐catalyzed steps (Kern et al., 2007) were normal in the TCA pathway in WT strain GXCR under Cu treatments, which was consistent with the results that the two enzymes were not affected under 3 mmol/L Cu (Figure 6a). Obviously, excessive depletion or low production of intermediates in the TCA chain such as fumarate, succinate, and citrate (Vuoristo, Mars, Sanders, Eggink, & Weusthuis, 2016), would lead to a risk of cyclic interruption of the TCA cycle. This may explain why the cycle initiating metabolite, citrate, was extremely increased in the later stage of 5 mmol/L Cu stress (Table 1), together with the increase in a portion of the citrate synthases under 3 mmol/L Cu (Figure 6a), which likely ensure normal TCA cycle activity. Together, these results not only increased the credibility of our protein files but also indirectly supported a conceptual model of high Cu sensitivity (Figure 10) and the above speculations that Cu stress mainly affects the later catalytic steps of glycolysis.

## Conflicts of Interest

The authors declare no conflict of interest.
