# Supplementary material for: A proteomic‐based investigation of potential copper‐responsive biomarkers: Proteins, conceptual networks, and metabolic pathways featuring Penicillium janthinellum from a heavy metal‐polluted ecological niche
Source: Microbiologyopen. 2017 May 9;6(4):e00485. doi: 10.1002/mbo3.485 (PMC5552966; doi:10.1002/mbo3.485)
Supplement: Supplementary file 1 [file MBO3-6-na-s001.zip › mbo3485-sup-0022-TableS1.docx]

**Table S1** The operating parameters of Mascot 2.3.0 version software for qualitative analysis of mass spectra

| **Name** | **Parameter option** |
| --- | --- |
| Fixed modification | Carbamidomethyl(C) |
| Variable modification | Oxidation(M), Gln→Pyro-Glu(N-term Q),  iTRAQ 8 plex(K), iTRAQ 8 plex(Y), iTRAQ  8 plex(N-term) |
| peptide tol. | 15 ppm |
| MS/MS tol | 20 mmu |
| Max missed cleavages 1 | Enzyme Trypsin |
| Database | Swissprot_Fungin |
