# Supplementary material for: A proteomic‐based investigation of potential copper‐responsive biomarkers: Proteins, conceptual networks, and metabolic pathways featuring Penicillium janthinellum from a heavy metal‐polluted ecological niche
Source: Microbiologyopen. 2017 May 9;6(4):e00485. doi: 10.1002/mbo3.485 (PMC5552966; doi:10.1002/mbo3.485)
Supplement: Supplementary file 1 [file MBO3-6-na-s001.zip › mbo3485-sup-0030-TableS9.docx]

| **Table S9** DPs co-regulated by 0, 0.5 and 3 mM Cu in EC-6 when comparing EC-6 vs. WT under the same treatments | | | | |
| --- | --- | --- | --- | --- |
| **Accession** | **Description** | **Expression fold in EC-6 at *p*< 0.05** | | |
|  |  | **0 mM Cu** | **0.5 mM Cu** | **3 mM Cu** |
| Q8SW28 | 14-3-3 protein homolog | 0.81 | 1.249 | 0.697 |
| P80346 | 2,3-dihydroxybenzoic acid decarboxylase | 1.529 | 0.787 | 0.144 |
| O93798 | 40S ribosomal protein S21 | 3.458 | 0.777 | 1.826 |
| Q8NKF4 | 60S ribosomal protein L3 | 0.781 | 6.61E-01 | 1.24E+00 |
| P16928 | Acetyl-coenzyme A synthetase | 2.64 | 0.648 | 0.245 |
| P36333 | Acetyl-coenzyme A synthetase | 1.455 | 1.291 | 0.826 |
| Q9UVZ8 | Actin | 0.742 | 2.075 | 1.305 |
| P08843 | Alcohol dehydrogenase 1 | 1.524 | 0.772 | 0.334 |
| P41751 | Aldehyde dehydrogenase | 1.299 | 0.785 | 0.579 |
| P32872 | Aldehyde dehydrogenase 2 | 2.165 | 0.593 | 0.389 |
| P22855 | Alpha-mannosidase | 0.609 | 0.719 | 0.741 |
| Q5B0J9 | ATP-dependent RNA helicase dbp2 | 1.561 | 0.429 | 1.537 |
| A6SFW7 | ATP-dependent RNA helicase dbp2 | 0.576 | 0.714 | 1.917 |
| B0YAK0 | Catalase-peroxidase | 1.642 | 0.722 | 0.76 |
| Q8NJN2 | Catalase-peroxidase | 1.491 | 0.661 | 0.626 |
| Q2H7U1 | Catalase-peroxidase | 1.259 | 0.68 | 0.599 |
| A4R5S9 | Catalase-peroxidase 1 | 3.179 | 0.684 | 0.68 |
| Q5AWS6 | Cell division control protein 48 | 0.683 | 1.285 | 1.3 |
| P30607 | Cytochrome P450 52A2 | 1.26 | 0.628 | 1.554 |
| B0XRV0 | Dipeptidyl-peptidase 5 | 0.796 | 1.47E+00 | 6.58E-01 |
| Q5AY89 | DNA damage-inducible protein 1 | 0.809 | 0.73 | 0.762 |
| Q4WJ02 | FACT complex subunit spt16 | 0.654 | 1.638 | 1.205 |
| O74254 | Glucoamylase 1 | 1.329 | 0.746 | 0.551 |
| P19089 | Glyceraldehyde-3-phosphate dehydrogenase | 0.579 | 1.36 | 0.599 |
| P55071 | Glyceraldehyde-3-phosphate dehydrogenase | 0.611 | 1.502 | 0.607 |
| Q96UF1 | Glyceraldehyde-3-phosphate dehydrogenase 3 | 0.645 | 1.583 | 0.729 |
| O94641 | Heat shock protein 104 | 0.819 | 1.284 | 1.477 |
| O43109 | Heat shock protein 90 homolog | 0.769 | 1.5 | 1.43 |
| P31540 | Heat shock protein hsp98 | 0.703 | 1.345 | 1.271 |
| A5DBG5 | Histone H2B.2 | 1.286 | 0.652 | 1.218 |
| P09322 | Histone H4 | 1.671 | 0.742 | 1.219 |
| O94225 | Homocitrate synthase | 0.763 | 1.213 | 1.353 |
| P54874 | Hydroxymethylglutaryl-CoA synthase | 0.618 | 0.495 | 1.554 |
| P28298 | Isocitrate lyase | 0.616 | 1.84 | 0.785 |
| A6R641 | Nascent polypeptide-associated complex subunit alpha | 0.368 | 1.281 | 0.821 |
| P07547 | Pentafunctional AROM polypeptide | 0.788 | 1.237 | 1.411 |
| Q01864 | pH-response transcription factor pacC/RIM101 | 1.656 | 1.426 | 1.332 |
| P62506 | Probable 60S ribosomal protein L44 | 1.211 | 1.211 | 0.807 |
| Q5Y223 | Probable electron transfer flavoprotein subunit alpha | 0.781 | 1.445 | 1.201 |
| P78827 | Probable ketol-acid reductoisomerase | 0.809 | 0.136 | 2.786 |
| P23639 | Proteasome component Y7 | 1.386 | 0.781 | 0.756 |
| Q09173 | Protein phosphatase 2C homolog 3 | 0.612 | 1.439 | 1.411 |
| Q9URY7 | Putative formamidase C869.04 | 1.366 | 0.677 | 0.402 |
| Q8X082 | Replication factor C subunit 5 | 0.819 | 1.394 | 1.295 |
| P35551 | rRNA 2'-O-methyltransferase fibrillarin | 0.614 | 1.883 | 1.292 |
| Q9P4R4 | Saccharopine dehydrogenase | 0.454 | 0.731 | 1.386 |
| Q6BRB7 | Sterol 24-C-methyltransferase | 0.833 | 0.825 | 1.816 |
| Q9Y8D9 | Sod [Cu-Zn] | 0.667 | 0.766 | 0.691 |
| A2QMY6 | Superoxide dismutase [Cu-Zn] | 0.489 | 0.8 | 0.731 |
| O42724 | Superoxide dismutase [Cu-Zn] 1 | 0.365 | 0.723 | 0.578 |
| P41799 | Tubulin beta chain | 0.762 | 0.829 | 1.458 |
| O74196 | Ubiquitin-conjugating enzyme E2-16 kDa | 0.806 | 0.828 | 1.405 |
| O14205 | Uncharacterized protein C5D6.13 | 0.638 | 3.251 | 1.495 |
| Q00511 | Uricase | 1.205 | 0.683 | 1.415 |
| O42926 | Vacuolar protein sorting-associated protein 13b | 1.562 | 1.332 | 0.77 |
