# Supplementary material for: A proteomic‐based investigation of potential copper‐responsive biomarkers: Proteins, conceptual networks, and metabolic pathways featuring Penicillium janthinellum from a heavy metal‐polluted ecological niche
Source: Microbiologyopen. 2017 May 9;6(4):e00485. doi: 10.1002/mbo3.485 (PMC5552966; doi:10.1002/mbo3.485)
Supplement: Supplementary file 1 [file MBO3-6-na-s001.zip › mbo3485-sup-0041-TableS20.docx]

| **Table S20** Pathways co-regulated under 0, 0.5 and 3 mM Cu in EC-6 when compared with EC-6 vs. WT under the same treatments | | | | |
| --- | --- | --- | --- | --- |
| **Pathway** | **The number of involved DEPs** | | |  |
|  | **0 mM Cu** | **0.5 mM Cu** | **3 mM Cu** | **Pathway ID** |
| [Tryptophan metabolism](file:///F:\李博士%20原桌面%20文件\学科学位、研究生管理\2011\许剑和董学伟博士研究课题\冯昕蛋白谱分析资料\Supplementary%20Tables\EC-WT%20PATHWAYS\Supplementary%20Table%20Pathways%20co-regulated%20by%20all%20conditions%20in%20EC-6%20as%20EC-6%20vs.%20WT%20.xlsx#Sheet1!gene1) | 8 | 7 | 9 | ko00380 |
| [Viral myocarditis](file:///F:\李博士%20原桌面%20文件\学科学位、研究生管理\2011\许剑和董学伟博士研究课题\冯昕蛋白谱分析资料\Supplementary%20Tables\EC-WT%20PATHWAYS\Supplementary%20Table%20Pathways%20co-regulated%20by%20all%20conditions%20in%20EC-6%20as%20EC-6%20vs.%20WT%20.xlsx#Sheet1!gene55) | 4 | 4 | 2 | ko05416 |
| [Vibrio cholerae infection](file:///F:\李博士%20原桌面%20文件\学科学位、研究生管理\2011\许剑和董学伟博士研究课题\冯昕蛋白谱分析资料\Supplementary%20Tables\EC-WT%20PATHWAYS\Supplementary%20Table%20Pathways%20co-regulated%20by%20all%20conditions%20in%20EC-6%20as%20EC-6%20vs.%20WT%20.xlsx#Sheet1!gene79) | 4 | 5 | 6 | ko05110 |
| [Valine, leucine and isoleucine degradation](file:///F:\李博士%20原桌面%20文件\学科学位、研究生管理\2011\许剑和董学伟博士研究课题\冯昕蛋白谱分析资料\Supplementary%20Tables\EC-WT%20PATHWAYS\Supplementary%20Table%20Pathways%20co-regulated%20by%20all%20conditions%20in%20EC-6%20as%20EC-6%20vs.%20WT%20.xlsx#Sheet1!gene5) | 6 | 4 | 5 | ko00280 |
| [Tyrosine metabolism](file:///F:\李博士%20原桌面%20文件\学科学位、研究生管理\2011\许剑和董学伟博士研究课题\冯昕蛋白谱分析资料\Supplementary%20Tables\EC-WT%20PATHWAYS\Supplementary%20Table%20Pathways%20co-regulated%20by%20all%20conditions%20in%20EC-6%20as%20EC-6%20vs.%20WT%20.xlsx#Sheet1!gene67) | 3 | 2 | 5 | ko00350 |
| [Toxoplasmosis](file:///F:\李博士%20原桌面%20文件\学科学位、研究生管理\2011\许剑和董学伟博士研究课题\冯昕蛋白谱分析资料\Supplementary%20Tables\EC-WT%20PATHWAYS\Supplementary%20Table%20Pathways%20co-regulated%20by%20all%20conditions%20in%20EC-6%20as%20EC-6%20vs.%20WT%20.xlsx#Sheet1!gene85) | 3 | 2 | 5 | ko05145 |
| [Tight junction](file:///F:\李博士%20原桌面%20文件\学科学位、研究生管理\2011\许剑和董学伟博士研究课题\冯昕蛋白谱分析资料\Supplementary%20Tables\EC-WT%20PATHWAYS\Supplementary%20Table%20Pathways%20co-regulated%20by%20all%20conditions%20in%20EC-6%20as%20EC-6%20vs.%20WT%20.xlsx#Sheet1!gene53) | 5 | 4 | 2 | ko04530 |
| [Systemic lupus erythematosus](file:///F:\李博士%20原桌面%20文件\学科学位、研究生管理\2011\许剑和董学伟博士研究课题\冯昕蛋白谱分析资料\Supplementary%20Tables\EC-WT%20PATHWAYS\Supplementary%20Table%20Pathways%20co-regulated%20by%20all%20conditions%20in%20EC-6%20as%20EC-6%20vs.%20WT%20.xlsx#Sheet1!gene29) | 5 | 6 | 3 | ko05322 |
| [Starch and sucrose metabolism](file:///F:\李博士%20原桌面%20文件\学科学位、研究生管理\2011\许剑和董学伟博士研究课题\冯昕蛋白谱分析资料\Supplementary%20Tables\EC-WT%20PATHWAYS\Supplementary%20Table%20Pathways%20co-regulated%20by%20all%20conditions%20in%20EC-6%20as%20EC-6%20vs.%20WT%20.xlsx#Sheet1!gene83) | 2 | 3 | 5 | ko00500 |
| [Spliceosome](file:///F:\李博士%20原桌面%20文件\学科学位、研究生管理\2011\许剑和董学伟博士研究课题\冯昕蛋白谱分析资料\Supplementary%20Tables\EC-WT%20PATHWAYS\Supplementary%20Table%20Pathways%20co-regulated%20by%20all%20conditions%20in%20EC-6%20as%20EC-6%20vs.%20WT%20.xlsx#Sheet1!gene81) | 7 | 5 | 9 | ko03040 |
| [Shigellosis](file:///F:\李博士%20原桌面%20文件\学科学位、研究生管理\2011\许剑和董学伟博士研究课题\冯昕蛋白谱分析资料\Supplementary%20Tables\EC-WT%20PATHWAYS\Supplementary%20Table%20Pathways%20co-regulated%20by%20all%20conditions%20in%20EC-6%20as%20EC-6%20vs.%20WT%20.xlsx#Sheet1!gene33) | 5 | 3 | 3 | ko05131 |
| [RNA transport](file:///F:\李博士%20原桌面%20文件\学科学位、研究生管理\2011\许剑和董学伟博士研究课题\冯昕蛋白谱分析资料\Supplementary%20Tables\EC-WT%20PATHWAYS\Supplementary%20Table%20Pathways%20co-regulated%20by%20all%20conditions%20in%20EC-6%20as%20EC-6%20vs.%20WT%20.xlsx#Sheet1!gene88) | 11 | 9 | 7 | ko03013 |
| [RNA degradation](file:///F:\李博士%20原桌面%20文件\学科学位、研究生管理\2011\许剑和董学伟博士研究课题\冯昕蛋白谱分析资料\Supplementary%20Tables\EC-WT%20PATHWAYS\Supplementary%20Table%20Pathways%20co-regulated%20by%20all%20conditions%20in%20EC-6%20as%20EC-6%20vs.%20WT%20.xlsx#Sheet1!gene76) | 5 | 2 | 2 | ko03018 |
| [Ribosome](file:///F:\李博士%20原桌面%20文件\学科学位、研究生管理\2011\许剑和董学伟博士研究课题\冯昕蛋白谱分析资料\Supplementary%20Tables\EC-WT%20PATHWAYS\Supplementary%20Table%20Pathways%20co-regulated%20by%20all%20conditions%20in%20EC-6%20as%20EC-6%20vs.%20WT%20.xlsx#Sheet1!gene4) | 21 | 11 | 8 | ko03010 |
| [Regulation of autophagy](file:///F:\李博士%20原桌面%20文件\学科学位、研究生管理\2011\许剑和董学伟博士研究课题\冯昕蛋白谱分析资料\Supplementary%20Tables\EC-WT%20PATHWAYS\Supplementary%20Table%20Pathways%20co-regulated%20by%20all%20conditions%20in%20EC-6%20as%20EC-6%20vs.%20WT%20.xlsx#Sheet1!gene25) | 3 | 2 | 3 | ko04140 |
| [Regulation of actin cytoskeleton](file:///F:\李博士%20原桌面%20文件\学科学位、研究生管理\2011\许剑和董学伟博士研究课题\冯昕蛋白谱分析资料\Supplementary%20Tables\EC-WT%20PATHWAYS\Supplementary%20Table%20Pathways%20co-regulated%20by%20all%20conditions%20in%20EC-6%20as%20EC-6%20vs.%20WT%20.xlsx#Sheet1!gene60) | 4 | 3 | 2 | ko04810 |
| [Pyruvate metabolism](file:///F:\李博士%20原桌面%20文件\学科学位、研究生管理\2011\许剑和董学伟博士研究课题\冯昕蛋白谱分析资料\Supplementary%20Tables\EC-WT%20PATHWAYS\Supplementary%20Table%20Pathways%20co-regulated%20by%20all%20conditions%20in%20EC-6%20as%20EC-6%20vs.%20WT%20.xlsx#Sheet1!gene2) | 14 | 10 | 12 | ko00620 |
| [Purine metabolism](file:///F:\李博士%20原桌面%20文件\学科学位、研究生管理\2011\许剑和董学伟博士研究课题\冯昕蛋白谱分析资料\Supplementary%20Tables\EC-WT%20PATHWAYS\Supplementary%20Table%20Pathways%20co-regulated%20by%20all%20conditions%20in%20EC-6%20as%20EC-6%20vs.%20WT%20.xlsx#Sheet1!gene80) | 5 | 6 | 4 | ko00230 |
| [Protein processing in endoplasmic reticulum](file:///F:\李博士%20原桌面%20文件\学科学位、研究生管理\2011\许剑和董学伟博士研究课题\冯昕蛋白谱分析资料\Supplementary%20Tables\EC-WT%20PATHWAYS\Supplementary%20Table%20Pathways%20co-regulated%20by%20all%20conditions%20in%20EC-6%20as%20EC-6%20vs.%20WT%20.xlsx#Sheet1!gene63) | 14 | 7 | 13 | ko04141 |
| [Proteasome](file:///F:\李博士%20原桌面%20文件\学科学位、研究生管理\2011\许剑和董学伟博士研究课题\冯昕蛋白谱分析资料\Supplementary%20Tables\EC-WT%20PATHWAYS\Supplementary%20Table%20Pathways%20co-regulated%20by%20all%20conditions%20in%20EC-6%20as%20EC-6%20vs.%20WT%20.xlsx#Sheet1!gene82) | 6 | 8 | 4 | ko03050 |
| [Propanoate metabolism](file:///F:\李博士%20原桌面%20文件\学科学位、研究生管理\2011\许剑和董学伟博士研究课题\冯昕蛋白谱分析资料\Supplementary%20Tables\EC-WT%20PATHWAYS\Supplementary%20Table%20Pathways%20co-regulated%20by%20all%20conditions%20in%20EC-6%20as%20EC-6%20vs.%20WT%20.xlsx#Sheet1!gene7) | 7 | 6 | 5 | ko00640 |
| [Prion diseases](file:///F:\李博士%20原桌面%20文件\学科学位、研究生管理\2011\许剑和董学伟博士研究课题\冯昕蛋白谱分析资料\Supplementary%20Tables\EC-WT%20PATHWAYS\Supplementary%20Table%20Pathways%20co-regulated%20by%20all%20conditions%20in%20EC-6%20as%20EC-6%20vs.%20WT%20.xlsx#Sheet1!gene3) | 5 | 3 | 5 | ko05020 |
| [Phototransduction - fly](file:///F:\李博士%20原桌面%20文件\学科学位、研究生管理\2011\许剑和董学伟博士研究课题\冯昕蛋白谱分析资料\Supplementary%20Tables\EC-WT%20PATHWAYS\Supplementary%20Table%20Pathways%20co-regulated%20by%20all%20conditions%20in%20EC-6%20as%20EC-6%20vs.%20WT%20.xlsx#Sheet1!gene31) | 5 | 3 | 2 | ko04745 |
| [Phenylpropanoid biosynthesis](file:///F:\李博士%20原桌面%20文件\学科学位、研究生管理\2011\许剑和董学伟博士研究课题\冯昕蛋白谱分析资料\Supplementary%20Tables\EC-WT%20PATHWAYS\Supplementary%20Table%20Pathways%20co-regulated%20by%20all%20conditions%20in%20EC-6%20as%20EC-6%20vs.%20WT%20.xlsx#Sheet1!gene18) | 4 | 5 | 5 | ko00940 |
| [Phenylalanine metabolism](file:///F:\李博士%20原桌面%20文件\学科学位、研究生管理\2011\许剑和董学伟博士研究课题\冯昕蛋白谱分析资料\Supplementary%20Tables\EC-WT%20PATHWAYS\Supplementary%20Table%20Pathways%20co-regulated%20by%20all%20conditions%20in%20EC-6%20as%20EC-6%20vs.%20WT%20.xlsx#Sheet1!gene20) | 5 | 5 | 7 | ko00360 |
| [Phagosome](file:///F:\李博士%20原桌面%20文件\学科学位、研究生管理\2011\许剑和董学伟博士研究课题\冯昕蛋白谱分析资料\Supplementary%20Tables\EC-WT%20PATHWAYS\Supplementary%20Table%20Pathways%20co-regulated%20by%20all%20conditions%20in%20EC-6%20as%20EC-6%20vs.%20WT%20.xlsx#Sheet1!gene70) | 7 | 8 | 11 | ko04145 |
| [Peroxisome](file:///F:\李博士%20原桌面%20文件\学科学位、研究生管理\2011\许剑和董学伟博士研究课题\冯昕蛋白谱分析资料\Supplementary%20Tables\EC-WT%20PATHWAYS\Supplementary%20Table%20Pathways%20co-regulated%20by%20all%20conditions%20in%20EC-6%20as%20EC-6%20vs.%20WT%20.xlsx#Sheet1!gene22) | 5 | 4 | 5 | ko04146 |
| [Pentose phosphate pathway](file:///F:\李博士%20原桌面%20文件\学科学位、研究生管理\2011\许剑和董学伟博士研究课题\冯昕蛋白谱分析资料\Supplementary%20Tables\EC-WT%20PATHWAYS\Supplementary%20Table%20Pathways%20co-regulated%20by%20all%20conditions%20in%20EC-6%20as%20EC-6%20vs.%20WT%20.xlsx#Sheet1!gene61) | 4 | 4 | 3 | ko00030 |
| [Pentose and glucuronate interconversions](file:///F:\李博士%20原桌面%20文件\学科学位、研究生管理\2011\许剑和董学伟博士研究课题\冯昕蛋白谱分析资料\Supplementary%20Tables\EC-WT%20PATHWAYS\Supplementary%20Table%20Pathways%20co-regulated%20by%20all%20conditions%20in%20EC-6%20as%20EC-6%20vs.%20WT%20.xlsx#Sheet1!gene10) | 3 | 2 | 3 | ko00040 |
| [Pathways in cancer](file:///F:\李博士%20原桌面%20文件\学科学位、研究生管理\2011\许剑和董学伟博士研究课题\冯昕蛋白谱分析资料\Supplementary%20Tables\EC-WT%20PATHWAYS\Supplementary%20Table%20Pathways%20co-regulated%20by%20all%20conditions%20in%20EC-6%20as%20EC-6%20vs.%20WT%20.xlsx#Sheet1!gene66) | 3 | 3 | 3 | ko05200 |
| [Pathogenic *Escherichia coli* infection](file:///F:\李博士%20原桌面%20文件\学科学位、研究生管理\2011\许剑和董学伟博士研究课题\冯昕蛋白谱分析资料\Supplementary%20Tables\EC-WT%20PATHWAYS\Supplementary%20Table%20Pathways%20co-regulated%20by%20all%20conditions%20in%20EC-6%20as%20EC-6%20vs.%20WT%20.xlsx#Sheet1!gene30) | 7 | 5 | 7 | ko05130 |
| [Oxidative phosphorylation](file:///F:\李博士%20原桌面%20文件\学科学位、研究生管理\2011\许剑和董学伟博士研究课题\冯昕蛋白谱分析资料\Supplementary%20Tables\EC-WT%20PATHWAYS\Supplementary%20Table%20Pathways%20co-regulated%20by%20all%20conditions%20in%20EC-6%20as%20EC-6%20vs.%20WT%20.xlsx#Sheet1!gene87) | 7 | 11 | 4 | ko00190 |
| [Oocyte meiosis](file:///F:\李博士%20原桌面%20文件\学科学位、研究生管理\2011\许剑和董学伟博士研究课题\冯昕蛋白谱分析资料\Supplementary%20Tables\EC-WT%20PATHWAYS\Supplementary%20Table%20Pathways%20co-regulated%20by%20all%20conditions%20in%20EC-6%20as%20EC-6%20vs.%20WT%20.xlsx#Sheet1!gene54) | 4 | 4 | 2 | ko04114 |
| [Nitrogen metabolism](file:///F:\李博士%20原桌面%20文件\学科学位、研究生管理\2011\许剑和董学伟博士研究课题\冯昕蛋白谱分析资料\Supplementary%20Tables\EC-WT%20PATHWAYS\Supplementary%20Table%20Pathways%20co-regulated%20by%20all%20conditions%20in%20EC-6%20as%20EC-6%20vs.%20WT%20.xlsx#Sheet1!gene8) | 5 | 3 | 2 | ko00910 |
| [Neurotrophin signaling pathway](file:///F:\李博士%20原桌面%20文件\学科学位、研究生管理\2011\许剑和董学伟博士研究课题\冯昕蛋白谱分析资料\Supplementary%20Tables\EC-WT%20PATHWAYS\Supplementary%20Table%20Pathways%20co-regulated%20by%20all%20conditions%20in%20EC-6%20as%20EC-6%20vs.%20WT%20.xlsx#Sheet1!gene27) | 4 | 2 | 3 | ko04722 |
| [mRNA surveillance pathway](file:///F:\李博士%20原桌面%20文件\学科学位、研究生管理\2011\许剑和董学伟博士研究课题\冯昕蛋白谱分析资料\Supplementary%20Tables\EC-WT%20PATHWAYS\Supplementary%20Table%20Pathways%20co-regulated%20by%20all%20conditions%20in%20EC-6%20as%20EC-6%20vs.%20WT%20.xlsx#Sheet1!gene93) | 2 | 4 | 2 | ko03015 |
| [Methane metabolism](file:///F:\李博士%20原桌面%20文件\学科学位、研究生管理\2011\许剑和董学伟博士研究课题\冯昕蛋白谱分析资料\Supplementary%20Tables\EC-WT%20PATHWAYS\Supplementary%20Table%20Pathways%20co-regulated%20by%20all%20conditions%20in%20EC-6%20as%20EC-6%20vs.%20WT%20.xlsx#Sheet1!gene59) | 8 | 9 | 12 | ko00680 |
| [MAPK signaling pathway](file:///F:\李博士%20原桌面%20文件\学科学位、研究生管理\2011\许剑和董学伟博士研究课题\冯昕蛋白谱分析资料\Supplementary%20Tables\EC-WT%20PATHWAYS\Supplementary%20Table%20Pathways%20co-regulated%20by%20all%20conditions%20in%20EC-6%20as%20EC-6%20vs.%20WT%20.xlsx#Sheet1!gene92) | 3 | 2 | 5 | ko04010 |
| [Lysine degradation](file:///F:\李博士%20原桌面%20文件\学科学位、研究生管理\2011\许剑和董学伟博士研究课题\冯昕蛋白谱分析资料\Supplementary%20Tables\EC-WT%20PATHWAYS\Supplementary%20Table%20Pathways%20co-regulated%20by%20all%20conditions%20in%20EC-6%20as%20EC-6%20vs.%20WT%20.xlsx#Sheet1!gene15) | 4 | 3 | 4 | ko00310 |
| [Lysine biosynthesis](file:///F:\李博士%20原桌面%20文件\学科学位、研究生管理\2011\许剑和董学伟博士研究课题\冯昕蛋白谱分析资料\Supplementary%20Tables\EC-WT%20PATHWAYS\Supplementary%20Table%20Pathways%20co-regulated%20by%20all%20conditions%20in%20EC-6%20as%20EC-6%20vs.%20WT%20.xlsx#Sheet1!gene46) | 2 | 2 | 2 | ko00300 |
| [Limonene and pinene degradation](file:///F:\李博士%20原桌面%20文件\学科学位、研究生管理\2011\许剑和董学伟博士研究课题\冯昕蛋白谱分析资料\Supplementary%20Tables\EC-WT%20PATHWAYS\Supplementary%20Table%20Pathways%20co-regulated%20by%20all%20conditions%20in%20EC-6%20as%20EC-6%20vs.%20WT%20.xlsx#Sheet1!gene12) | 3 | 2 | 3 | ko00903 |
| [Leukocyte transendothelial migration](file:///F:\李博士%20原桌面%20文件\学科学位、研究生管理\2011\许剑和董学伟博士研究课题\冯昕蛋白谱分析资料\Supplementary%20Tables\EC-WT%20PATHWAYS\Supplementary%20Table%20Pathways%20co-regulated%20by%20all%20conditions%20in%20EC-6%20as%20EC-6%20vs.%20WT%20.xlsx#Sheet1!gene32) | 5 | 3 | 3 | ko04670 |
| [Insulin signaling pathway](file:///F:\李博士%20原桌面%20文件\学科学位、研究生管理\2011\许剑和董学伟博士研究课题\冯昕蛋白谱分析资料\Supplementary%20Tables\EC-WT%20PATHWAYS\Supplementary%20Table%20Pathways%20co-regulated%20by%20all%20conditions%20in%20EC-6%20as%20EC-6%20vs.%20WT%20.xlsx#Sheet1!gene72) | 3 | 2 | 4 | ko04910 |
| [Hypertrophic cardiomyopathy (HCM)](file:///F:\李博士%20原桌面%20文件\学科学位、研究生管理\2011\许剑和董学伟博士研究课题\冯昕蛋白谱分析资料\Supplementary%20Tables\EC-WT%20PATHWAYS\Supplementary%20Table%20Pathways%20co-regulated%20by%20all%20conditions%20in%20EC-6%20as%20EC-6%20vs.%20WT%20.xlsx#Sheet1!gene35) | 4 | 3 | 2 | ko05410 |
| [Huntington's disease](file:///F:\李博士%20原桌面%20文件\学科学位、研究生管理\2011\许剑和董学伟博士研究课题\冯昕蛋白谱分析资料\Supplementary%20Tables\EC-WT%20PATHWAYS\Supplementary%20Table%20Pathways%20co-regulated%20by%20all%20conditions%20in%20EC-6%20as%20EC-6%20vs.%20WT%20.xlsx#Sheet1!gene71) | 11 | 13 | 4 | ko05016 |
| [Histidine metabolism](file:///F:\李博士%20原桌面%20文件\学科学位、研究生管理\2011\许剑和董学伟博士研究课题\冯昕蛋白谱分析资料\Supplementary%20Tables\EC-WT%20PATHWAYS\Supplementary%20Table%20Pathways%20co-regulated%20by%20all%20conditions%20in%20EC-6%20as%20EC-6%20vs.%20WT%20.xlsx#Sheet1!gene6) | 4 | 3 | 3 | ko00340 |
| [Hepatitis C](file:///F:\李博士%20原桌面%20文件\学科学位、研究生管理\2011\许剑和董学伟博士研究课题\冯昕蛋白谱分析资料\Supplementary%20Tables\EC-WT%20PATHWAYS\Supplementary%20Table%20Pathways%20co-regulated%20by%20all%20conditions%20in%20EC-6%20as%20EC-6%20vs.%20WT%20.xlsx#Sheet1!gene78) | 2 | 2 | 2 | ko05160 |
| [Glyoxylate and dicarboxylate metabolism](file:///F:\李博士%20原桌面%20文件\学科学位、研究生管理\2011\许剑和董学伟博士研究课题\冯昕蛋白谱分析资料\Supplementary%20Tables\EC-WT%20PATHWAYS\Supplementary%20Table%20Pathways%20co-regulated%20by%20all%20conditions%20in%20EC-6%20as%20EC-6%20vs.%20WT%20.xlsx#Sheet1!gene86) | 3 | 6 | 7 | ko00630 |

| **Table S20***Continued* | | | | |
| --- | --- | --- | --- | --- |
| **Pathway** | **The number of involved DEPs** | | |  |
|  | **0 mM Cu** | **0.5 mM Cu** | **3 mM Cu** | **Pathway ID** |
| [Glycolysis / Gluconeogenesis](file:///F:\李博士%20原桌面%20文件\学科学位、研究生管理\2011\许剑和董学伟博士研究课题\冯昕蛋白谱分析资料\Supplementary%20Tables\EC-WT%20PATHWAYS\Supplementary%20Table%20Pathways%20co-regulated%20by%20all%20conditions%20in%20EC-6%20as%20EC-6%20vs.%20WT%20.xlsx#Sheet1!gene57) | 19 | 13 | 23 | ko00010 |
| [Glycine, serine and threonine metabolism](file:///F:\李博士%20原桌面%20文件\学科学位、研究生管理\2011\许剑和董学伟博士研究课题\冯昕蛋白谱分析资料\Supplementary%20Tables\EC-WT%20PATHWAYS\Supplementary%20Table%20Pathways%20co-regulated%20by%20all%20conditions%20in%20EC-6%20as%20EC-6%20vs.%20WT%20.xlsx#Sheet1!gene28) | 4 | 2 | 3 | ko00260 |
| [Glycerolipid metabolism](file:///F:\李博士%20原桌面%20文件\学科学位、研究生管理\2011\许剑和董学伟博士研究课题\冯昕蛋白谱分析资料\Supplementary%20Tables\EC-WT%20PATHWAYS\Supplementary%20Table%20Pathways%20co-regulated%20by%20all%20conditions%20in%20EC-6%20as%20EC-6%20vs.%20WT%20.xlsx#Sheet1!gene11) | 3 | 2 | 3 | ko00561 |
| [Gap junction](file:///F:\李博士%20原桌面%20文件\学科学位、研究生管理\2011\许剑和董学伟博士研究课题\冯昕蛋白谱分析资料\Supplementary%20Tables\EC-WT%20PATHWAYS\Supplementary%20Table%20Pathways%20co-regulated%20by%20all%20conditions%20in%20EC-6%20as%20EC-6%20vs.%20WT%20.xlsx#Sheet1!gene65) | 3 | 2 | 5 | ko04540 |
| [Galactose metabolism](file:///F:\李博士%20原桌面%20文件\学科学位、研究生管理\2011\许剑和董学伟博士研究课题\冯昕蛋白谱分析资料\Supplementary%20Tables\EC-WT%20PATHWAYS\Supplementary%20Table%20Pathways%20co-regulated%20by%20all%20conditions%20in%20EC-6%20as%20EC-6%20vs.%20WT%20.xlsx#Sheet1!gene77) | 2 | 2 | 4 | ko00052 |
| [Focal adhesion](file:///F:\李博士%20原桌面%20文件\学科学位、研究生管理\2011\许剑和董学伟博士研究课题\冯昕蛋白谱分析资料\Supplementary%20Tables\EC-WT%20PATHWAYS\Supplementary%20Table%20Pathways%20co-regulated%20by%20all%20conditions%20in%20EC-6%20as%20EC-6%20vs.%20WT%20.xlsx#Sheet1!gene62) | 4 | 3 | 2 | ko04510 |
| [Fatty acid metabolism](file:///F:\李博士%20原桌面%20文件\学科学位、研究生管理\2011\许剑和董学伟博士研究课题\冯昕蛋白谱分析资料\Supplementary%20Tables\EC-WT%20PATHWAYS\Supplementary%20Table%20Pathways%20co-regulated%20by%20all%20conditions%20in%20EC-6%20as%20EC-6%20vs.%20WT%20.xlsx#Sheet1!gene9) | 5 | 3 | 6 | ko00071 |
| [Endocytosis](file:///F:\李博士%20原桌面%20文件\学科学位、研究生管理\2011\许剑和董学伟博士研究课题\冯昕蛋白谱分析资料\Supplementary%20Tables\EC-WT%20PATHWAYS\Supplementary%20Table%20Pathways%20co-regulated%20by%20all%20conditions%20in%20EC-6%20as%20EC-6%20vs.%20WT%20.xlsx#Sheet1!gene89) | 3 | 3 | 5 | ko04144 |
| [Dilated cardiomyopathy](file:///F:\李博士%20原桌面%20文件\学科学位、研究生管理\2011\许剑和董学伟博士研究课题\冯昕蛋白谱分析资料\Supplementary%20Tables\EC-WT%20PATHWAYS\Supplementary%20Table%20Pathways%20co-regulated%20by%20all%20conditions%20in%20EC-6%20as%20EC-6%20vs.%20WT%20.xlsx#Sheet1!gene38) | 4 | 3 | 2 | ko05414 |
| [Citrate cycle (TCA cycle)](file:///F:\李博士%20原桌面%20文件\学科学位、研究生管理\2011\许剑和董学伟博士研究课题\冯昕蛋白谱分析资料\Supplementary%20Tables\EC-WT%20PATHWAYS\Supplementary%20Table%20Pathways%20co-regulated%20by%20all%20conditions%20in%20EC-6%20as%20EC-6%20vs.%20WT%20.xlsx#Sheet1!gene58) | 8 | 6 | 7 | ko00020 |
| [Chloroalkane and chloroalkene degradation](file:///F:\李博士%20原桌面%20文件\学科学位、研究生管理\2011\许剑和董学伟博士研究课题\冯昕蛋白谱分析资料\Supplementary%20Tables\EC-WT%20PATHWAYS\Supplementary%20Table%20Pathways%20co-regulated%20by%20all%20conditions%20in%20EC-6%20as%20EC-6%20vs.%20WT%20.xlsx#Sheet1!gene23) | 5 | 3 | 7 | ko00625 |
| [Cell cycle](file:///F:\李博士%20原桌面%20文件\学科学位、研究生管理\2011\许剑和董学伟博士研究课题\冯昕蛋白谱分析资料\Supplementary%20Tables\EC-WT%20PATHWAYS\Supplementary%20Table%20Pathways%20co-regulated%20by%20all%20conditions%20in%20EC-6%20as%20EC-6%20vs.%20WT%20.xlsx#Sheet1!gene51) | 2 | 2 | 2 | ko04110 |
| [Carbon fixation in prokaryotes](file:///F:\李博士%20原桌面%20文件\学科学位、研究生管理\2011\许剑和董学伟博士研究课题\冯昕蛋白谱分析资料\Supplementary%20Tables\EC-WT%20PATHWAYS\Supplementary%20Table%20Pathways%20co-regulated%20by%20all%20conditions%20in%20EC-6%20as%20EC-6%20vs.%20WT%20.xlsx#Sheet1!gene39) | 3 | 4 | 3 | ko00720 |
| [beta-Alanine metabolism](file:///F:\李博士%20原桌面%20文件\学科学位、研究生管理\2011\许剑和董学伟博士研究课题\冯昕蛋白谱分析资料\Supplementary%20Tables\EC-WT%20PATHWAYS\Supplementary%20Table%20Pathways%20co-regulated%20by%20all%20conditions%20in%20EC-6%20as%20EC-6%20vs.%20WT%20.xlsx#Sheet1!gene37) | 4 | 2 | 6 | ko00410 |
| [Bacterial invasion of epithelial cells](file:///F:\李博士%20原桌面%20文件\学科学位、研究生管理\2011\许剑和董学伟博士研究课题\冯昕蛋白谱分析资料\Supplementary%20Tables\EC-WT%20PATHWAYS\Supplementary%20Table%20Pathways%20co-regulated%20by%20all%20conditions%20in%20EC-6%20as%20EC-6%20vs.%20WT%20.xlsx#Sheet1!gene56) | 4 | 3 | 2 | ko05100 |
| [Ascorbate and aldarate metabolism](file:///F:\李博士%20原桌面%20文件\学科学位、研究生管理\2011\许剑和董学伟博士研究课题\冯昕蛋白谱分析资料\Supplementary%20Tables\EC-WT%20PATHWAYS\Supplementary%20Table%20Pathways%20co-regulated%20by%20all%20conditions%20in%20EC-6%20as%20EC-6%20vs.%20WT%20.xlsx#Sheet1!gene13) | 3 | 2 | 3 | ko00053 |
| [Arrhythmogenic right ventricular cardiomyopathy (ARVC)](file:///F:\李博士%20原桌面%20文件\学科学位、研究生管理\2011\许剑和董学伟博士研究课题\冯昕蛋白谱分析资料\Supplementary%20Tables\EC-WT%20PATHWAYS\Supplementary%20Table%20Pathways%20co-regulated%20by%20all%20conditions%20in%20EC-6%20as%20EC-6%20vs.%20WT%20.xlsx#Sheet1!gene34) | 4 | 3 | 2 | ko05412 |
| [Arginine and proline metabolism](file:///F:\李博士%20原桌面%20文件\学科学位、研究生管理\2011\许剑和董学伟博士研究课题\冯昕蛋白谱分析资料\Supplementary%20Tables\EC-WT%20PATHWAYS\Supplementary%20Table%20Pathways%20co-regulated%20by%20all%20conditions%20in%20EC-6%20as%20EC-6%20vs.%20WT%20.xlsx#Sheet1!gene52) | 5 | 5 | 7 | ko00330 |
| [Antigen processing and presentation](file:///F:\李博士%20原桌面%20文件\学科学位、研究生管理\2011\许剑和董学伟博士研究课题\冯昕蛋白谱分析资料\Supplementary%20Tables\EC-WT%20PATHWAYS\Supplementary%20Table%20Pathways%20co-regulated%20by%20all%20conditions%20in%20EC-6%20as%20EC-6%20vs.%20WT%20.xlsx#Sheet1!gene74) | 6 | 2 | 7 | ko04612 |
| [Amyotrophic lateral sclerosis (ALS)](file:///F:\李博士%20原桌面%20文件\学科学位、研究生管理\2011\许剑和董学伟博士研究课题\冯昕蛋白谱分析资料\Supplementary%20Tables\EC-WT%20PATHWAYS\Supplementary%20Table%20Pathways%20co-regulated%20by%20all%20conditions%20in%20EC-6%20as%20EC-6%20vs.%20WT%20.xlsx#Sheet1!gene21) | 5 | 5 | 5 | ko05014 |
| [Amino sugar and nucleotide sugar metabolism](file:///F:\李博士%20原桌面%20文件\学科学位、研究生管理\2011\许剑和董学伟博士研究课题\冯昕蛋白谱分析资料\Supplementary%20Tables\EC-WT%20PATHWAYS\Supplementary%20Table%20Pathways%20co-regulated%20by%20all%20conditions%20in%20EC-6%20as%20EC-6%20vs.%20WT%20.xlsx#Sheet1!gene90) | 3 | 4 | 5 | ko00520 |
| [Alzheimer's disease](file:///F:\李博士%20原桌面%20文件\学科学位、研究生管理\2011\许剑和董学伟博士研究课题\冯昕蛋白谱分析资料\Supplementary%20Tables\EC-WT%20PATHWAYS\Supplementary%20Table%20Pathways%20co-regulated%20by%20all%20conditions%20in%20EC-6%20as%20EC-6%20vs.%20WT%20.xlsx#Sheet1!gene84) | 9 | 12 | 4 | ko05010 |
| [Alanine, aspartate and glutamate metabolism](file:///F:\李博士%20原桌面%20文件\学科学位、研究生管理\2011\许剑和董学伟博士研究课题\冯昕蛋白谱分析资料\Supplementary%20Tables\EC-WT%20PATHWAYS\Supplementary%20Table%20Pathways%20co-regulated%20by%20all%20conditions%20in%20EC-6%20as%20EC-6%20vs.%20WT%20.xlsx#Sheet1!gene75) | 4 | 3 | 2 | ko00250 |
| [Adherens junction](file:///F:\李博士%20原桌面%20文件\学科学位、研究生管理\2011\许剑和董学伟博士研究课题\冯昕蛋白谱分析资料\Supplementary%20Tables\EC-WT%20PATHWAYS\Supplementary%20Table%20Pathways%20co-regulated%20by%20all%20conditions%20in%20EC-6%20as%20EC-6%20vs.%20WT%20.xlsx#Sheet1!gene36) | 4 | 3 | 2 | ko04520 |
