# Supplementary material for: A proteomic‐based investigation of potential copper‐responsive biomarkers: Proteins, conceptual networks, and metabolic pathways featuring Penicillium janthinellum from a heavy metal‐polluted ecological niche
Source: Microbiologyopen. 2017 May 9;6(4):e00485. doi: 10.1002/mbo3.485 (PMC5552966; doi:10.1002/mbo3.485)
Supplement: Supplementary file 1 [file MBO3-6-na-s001.zip › mbo3485-sup-0042-TableS21.docx]

| **Table S21** Pathways regulated only under specific conditions in EC-6 when compared with EC-6 vs. WT under the same treatments | | | | |
| --- | --- | --- | --- | --- |
| **Pathway** | **The number of involved DEPs** | | |  |
|  | **0 mM Cu** | **0.5 mM Cu** | **3 mM Cu** | **Pathway ID** |
| [Aminoacyl-tRNA biosynthesis](file:///F:\李博士%20原桌面%20文件\学科学位、研究生管理\2011\许剑和董学伟博士研究课题\冯昕蛋白谱分析资料\Supplementary%20Tables\EC-WT%20PATHWAYS\Supplementary%20Table%20Pathways%20only%20regulated%20by%20specific%20conditions%20in%20EC-6%20as%20EC-6%20vs.%20WT%20.xlsx#RANGE!gene47) | 2 | / | / | ko00970 |
|  | Alanyl-tRNA synthetase | | | |
|  | Seryl-tRNA synthetase | | | |
|  |  | | | |
| [Chagas disease (American trypanosomiasis)](file:///F:\李博士%20原桌面%20文件\学科学位、研究生管理\2011\许剑和董学伟博士研究课题\冯昕蛋白谱分析资料\Supplementary%20Tables\EC-WT%20PATHWAYS\Supplementary%20Table%20Pathways%20only%20regulated%20by%20specific%20conditions%20in%20EC-6%20as%20EC-6%20vs.%20WT%20.xlsx#RANGE!gene45) | 2 | / | / | ko05142 |
|  | Protein phosphatase PP2A regulatory subunit A | | | |
|  | Mitogen-activated protein kinase hog1 | | | |
|  |  | | | |
| [GnRH signaling pathway](file:///F:\李博士%20原桌面%20文件\学科学位、研究生管理\2011\许剑和董学伟博士研究课题\冯昕蛋白谱分析资料\Supplementary%20Tables\EC-WT%20PATHWAYS\Supplementary%20Table%20Pathways%20only%20regulated%20by%20specific%20conditions%20in%20EC-6%20as%20EC-6%20vs.%20WT%20.xlsx#RANGE!gene49) | 2 | / | / | ko04912 |
|  | Calmodulin |  |  |  |
|  | Mitogen-activated protein kinase hog1 | | | |
|  |  |  |  |  |
| [Cardiac muscle contraction](file:///F:\李博士%20原桌面%20文件\学科学位、研究生管理\2011\许剑和董学伟博士研究课题\冯昕蛋白谱分析资料\Supplementary%20Tables\EC-WT%20PATHWAYS\Supplementary%20Table%20Pathways%20only%20regulated%20by%20specific%20conditions%20in%20EC-6%20as%20EC-6%20vs.%20WT%20.xlsx#RANGE!gene11) | / | 2 | / | ko04260 |
|  |  | Cytochrome c oxidase polypeptide5 | | |
|  |  | Cytochromec1 | | |
|  |  |  | | |
| [Cell cycle - yeast](file:///F:\李博士%20原桌面%20文件\学科学位、研究生管理\2011\许剑和董学伟博士研究课题\冯昕蛋白谱分析资料\Supplementary%20Tables\EC-WT%20PATHWAYS\Supplementary%20Table%20Pathways%20only%20regulated%20by%20specific%20conditions%20in%20EC-6%20as%20EC-6%20vs.%20WT%20.xlsx#RANGE!gene31) | / | 2 | / | ko04111 |
|  |  | Cell division control protein 2 | | |
|  |  | Protein phosphatase PP2A regulatory  subunit A | | |
|  |  | | | |
| [Colorectal cancer](file:///F:\李博士%20原桌面%20文件\学科学位、研究生管理\2011\许剑和董学伟博士研究课题\冯昕蛋白谱分析资料\Supplementary%20Tables\EC-WT%20PATHWAYS\Supplementary%20Table%20Pathways%20only%20regulated%20by%20specific%20conditions%20in%20EC-6%20as%20EC-6%20vs.%20WT%20.xlsx#RANGE!gene9) | / | 2 | / | ko05210 |
|  |  | Cytochrome c | | |
|  |  | DNA mismatch repair protein MSH3 | | |
|  |  |  | | |
| [Fatty acid biosynthesis](file:///F:\李博士%20原桌面%20文件\学科学位、研究生管理\2011\许剑和董学伟博士研究课题\冯昕蛋白谱分析资料\Supplementary%20Tables\EC-WT%20PATHWAYS\Supplementary%20Table%20Pathways%20only%20regulated%20by%20specific%20conditions%20in%20EC-6%20as%20EC-6%20vs.%20WT%20.xlsx#RANGE!gene13) | / | 3 | / | ko00061 |
|  |  | Acetyl-CoA carboxylase | | |
|  |  | Fatty acid synthase subunit alpha | | |
|  |  | Putative fatty acid synthase subunit TOXC | | |
|  |  |  | | |
| [Meiosis - yeast](file:///F:\李博士%20原桌面%20文件\学科学位、研究生管理\2011\许剑和董学伟博士研究课题\冯昕蛋白谱分析资料\Supplementary%20Tables\EC-WT%20PATHWAYS\Supplementary%20Table%20Pathways%20only%20regulated%20by%20specific%20conditions%20in%20EC-6%20as%20EC-6%20vs.%20WT%20.xlsx#RANGE!gene46) | / | 2 | / | ko04113 |
|  |  | Cell division control protein 2 | | |
|  |  | Protein phosphatase PP2A regulatory subunit A | | |
|  |  |  | | |
| [Ubiquitin mediated proteolysis](file:///F:\李博士%20原桌面%20文件\学科学位、研究生管理\2011\许剑和董学伟博士研究课题\冯昕蛋白谱分析资料\Supplementary%20Tables\EC-WT%20PATHWAYS\Supplementary%20Table%20Pathways%20only%20regulated%20by%20specific%20conditions%20in%20EC-6%20as%20EC-6%20vs.%20WT%20.xlsx#RANGE!gene43) | / | 2 | / | ko04120 |
|  |  | Ubiquitin-conjugating enzyme E2-16 kDa | | |
|  |  | Ubiquitin-activating enzyme E1 | | |
|  |  |  | | |
| [Wnt signaling pathway](file:///F:\李博士%20原桌面%20文件\学科学位、研究生管理\2011\许剑和董学伟博士研究课题\冯昕蛋白谱分析资料\Supplementary%20Tables\EC-WT%20PATHWAYS\Supplementary%20Table%20Pathways%20only%20regulated%20by%20specific%20conditions%20in%20EC-6%20as%20EC-6%20vs.%20WT%20.xlsx#RANGE!gene12) | / | 4 | / | ko04310 |
|  |  | Serine/threonine-protein phosphatase 2B catalytic subunit | | |
|  |  | Protein phosphatase PP2A regulatory subunit A | | |
|  |  | RuvB-like helicase 1[*Yarrowia lipolytica*] | | |
|  |  | RuvB-like helicase 1[*Emericella nidulans*] | | |

| **Table S21** *Continued* | | | | |
| --- | --- | --- | --- | --- |
| **Pathway** | **The number of involved DEPs** | | |  |
|  | **0 mM Cu** | **0.5 mM Cu** | **3 mM Cu** | **Pathway ID** |
| [Carbohydrate digestion and absorption](file:///F:\李博士%20原桌面%20文件\学科学位、研究生管理\2011\许剑和董学伟博士研究课题\冯昕蛋白谱分析资料\Supplementary%20Tables\EC-WT%20PATHWAYS\Supplementary%20Table%20Pathways%20only%20regulated%20by%20specific%20conditions%20in%20EC-6%20as%20EC-6%20vs.%20WT%20.xlsx#RANGE!gene35) | / | / | 2 | ko04973 |
|  |  |  | Acetyl-coenzyme A synthetase | |
|  |  |  | Isocitrate dehydrogenase [NADP] | |
|  |  |  |  | |
| [Butanoate metabolism](file:///F:\李博士%20原桌面%20文件\学科学位、研究生管理\2011\许剑和董学伟博士研究课题\冯昕蛋白谱分析资料\Supplementary%20Tables\EC-WT%20PATHWAYS\Supplementary%20Table%20Pathways%20only%20regulated%20by%20specific%20conditions%20in%20EC-6%20as%20EC-6%20vs.%20WT%20.xlsx#RANGE!gene64) | / | / | 2 | ko00650 |
|  |  |  | Hydroxymethylglutaryl-CoA synthase | |
|  |  |  | Peroxisomal hydratase-dehydrogenase-epimerase | |
|  |  |  |  | |
| [Butirosin and neomycin biosynthesis](file:///F:\李博士%20原桌面%20文件\学科学位、研究生管理\2011\许剑和董学伟博士研究课题\冯昕蛋白谱分析资料\Supplementary%20Tables\EC-WT%20PATHWAYS\Supplementary%20Table%20Pathways%20only%20regulated%20by%20specific%20conditions%20in%20EC-6%20as%20EC-6%20vs.%20WT%20.xlsx#RANGE!gene36) | / | / | 2 | ko00524 |
|  |  |  | Glucokinase | |
|  |  |  | Hexokinase | |
|  |  |  |  | |
| [Isoquinoline alkaloid biosynthesis](file:///F:\李博士%20原桌面%20文件\学科学位、研究生管理\2011\许剑和董学伟博士研究课题\冯昕蛋白谱分析资料\Supplementary%20Tables\EC-WT%20PATHWAYS\Supplementary%20Table%20Pathways%20only%20regulated%20by%20specific%20conditions%20in%20EC-6%20as%20EC-6%20vs.%20WT%20.xlsx#RANGE!gene33) | / | / | 2 | ko00950 |
|  |  |  | Copper amine oxidase 1 | |
|  |  |  | Peroxisomal primary amine oxidase | |
|  |  |  |  | |
| [Fructose and mannose metabolism](file:///F:\李博士%20原桌面%20文件\学科学位、研究生管理\2011\许剑和董学伟博士研究课题\冯昕蛋白谱分析资料\Supplementary%20Tables\EC-WT%20PATHWAYS\Supplementary%20Table%20Pathways%20only%20regulated%20by%20specific%20conditions%20in%20EC-6%20as%20EC-6%20vs.%20WT%20.xlsx#RANGE!gene51) | / | / | 5 | ko00051 |
|  |  |  | Glucokinase | |
|  |  |  | Hexokinase | |
|  |  |  | Mannose-1-phosphate guanyltransferase | |
|  |  |  | Mannitol-1-phosphate 5-dehydrogenase | |
|  |  |  | Peroxisomal hydratase-dehydrogenase-epimerase | |
|  |  |  |  | |
| [Phenylalanine, tyrosine and tryptophan biosynthesis](file:///F:\李博士%20原桌面%20文件\学科学位、研究生管理\2011\许剑和董学伟博士研究课题\冯昕蛋白谱分析资料\Supplementary%20Tables\EC-WT%20PATHWAYS\Supplementary%20Table%20Pathways%20only%20regulated%20by%20specific%20conditions%20in%20EC-6%20as%20EC-6%20vs.%20WT%20.xlsx#RANGE!gene72) | / | / | 2 | ko00400 |
|  |  |  | Phospho-2-dehydro-3-deoxyheptonate aldolase | |
|  |  |  | Pentafunctional AROM polypeptide | |
|  |  |  |  | |
| [Streptomycin biosynthesis](file:///F:\李博士%20原桌面%20文件\学科学位、研究生管理\2011\许剑和董学伟博士研究课题\冯昕蛋白谱分析资料\Supplementary%20Tables\EC-WT%20PATHWAYS\Supplementary%20Table%20Pathways%20only%20regulated%20by%20specific%20conditions%20in%20EC-6%20as%20EC-6%20vs.%20WT%20.xlsx#RANGE!gene39) | / | / | 3 | ko00521 |
|  |  |  | Phosphoglucomutase | |
|  |  |  | Glucokinase | |
|  |  |  | Hexokinase | |
|  |  |  |  | |
| [Tropane, piperidine and pyridine alkaloid biosynthesis](file:///F:\李博士%20原桌面%20文件\学科学位、研究生管理\2011\许剑和董学伟博士研究课题\冯昕蛋白谱分析资料\Supplementary%20Tables\EC-WT%20PATHWAYS\Supplementary%20Table%20Pathways%20only%20regulated%20by%20specific%20conditions%20in%20EC-6%20as%20EC-6%20vs.%20WT%20.xlsx#RANGE!gene32) | / | / | 2 | ko00960 |
|  |  |  | Copper amine oxidase 1 | |
|  |  |  | Peroxisomal primary amine oxidase | |
|  |  |  |  | |
| [Type II diabetes mellitus](file:///F:\李博士%20原桌面%20文件\学科学位、研究生管理\2011\许剑和董学伟博士研究课题\冯昕蛋白谱分析资料\Supplementary%20Tables\EC-WT%20PATHWAYS\Supplementary%20Table%20Pathways%20only%20regulated%20by%20specific%20conditions%20in%20EC-6%20as%20EC-6%20vs.%20WT%20.xlsx#RANGE!gene46) | / | / | 3 | ko04930 |
|  |  |  | Pyruvate kinase | |
|  |  |  | Glucokinase | |
|  |  |  | Hexokinase | |
