# Supplementary figures and images for: A proteomic‐based investigation of potential copper‐responsive biomarkers: Proteins, conceptual networks, and metabolic pathways featuring Penicillium janthinellum from a heavy metal‐polluted ecological niche
Source: Microbiologyopen. 2017 May 9;6(4):e00485. doi: 10.1002/mbo3.485 (PMC5552966; doi:10.1002/mbo3.485)

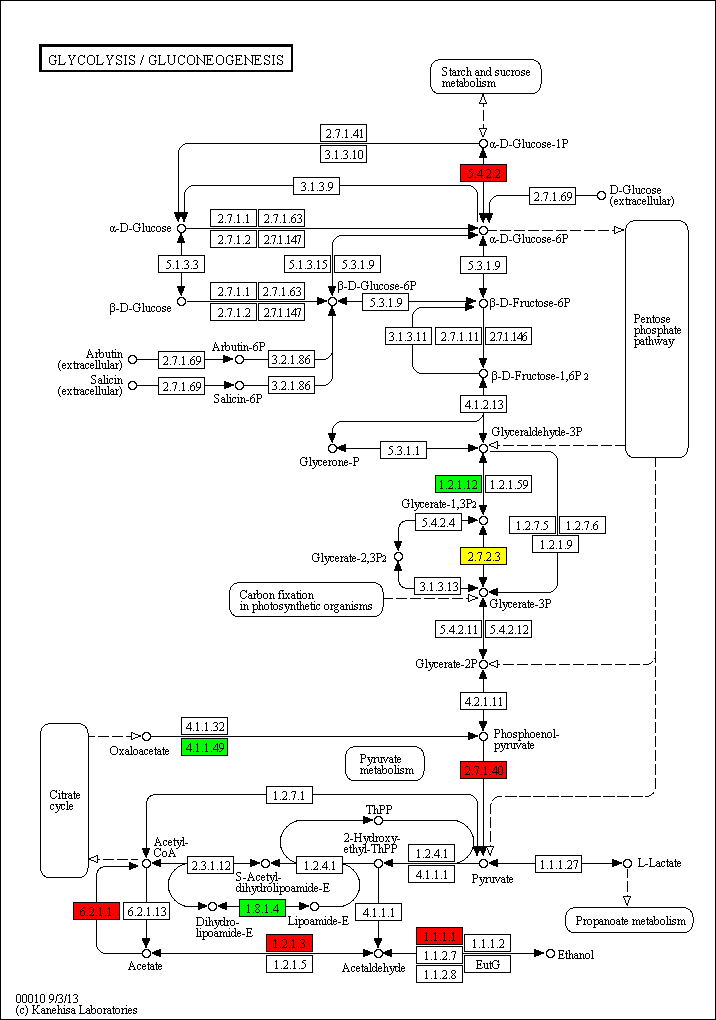

Supplement: Supplementary file 1 [file MBO3-6-na-s001.zip › mbo3485-sup-0001-FigS1.jpg]

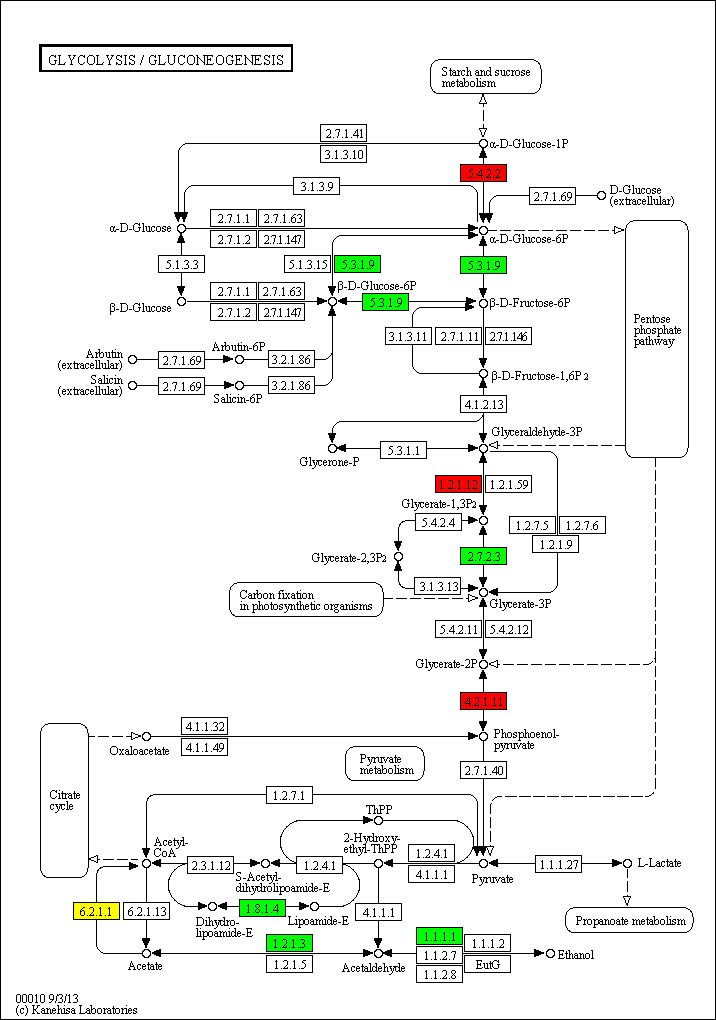

Supplement: Supplementary file 1 [file MBO3-6-na-s001.zip › mbo3485-sup-0002-FigS2.jpg]

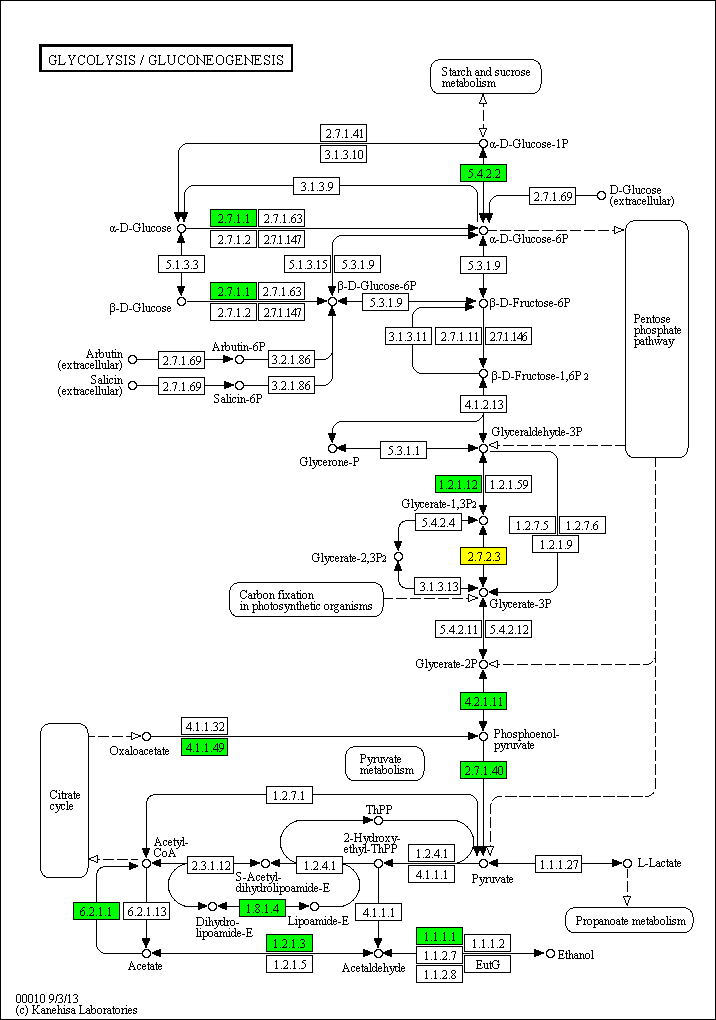

Supplement: Supplementary file 1 [file MBO3-6-na-s001.zip › mbo3485-sup-0003-FigS3.jpg]

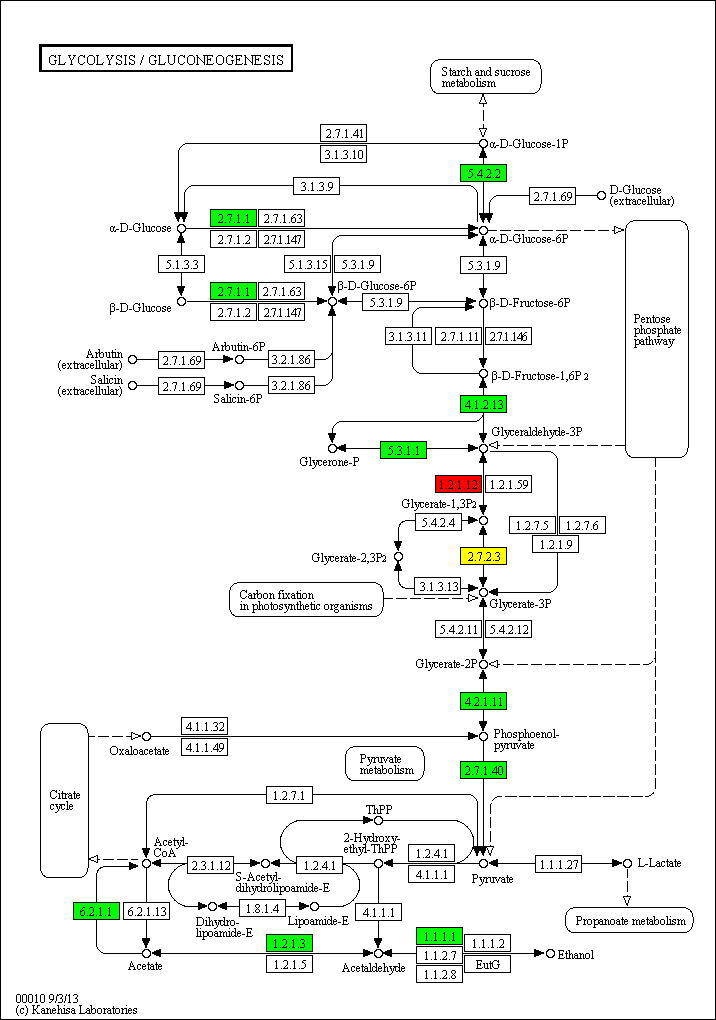

Supplement: Supplementary file 1 [file MBO3-6-na-s001.zip › mbo3485-sup-0004-FigS4.jpg]

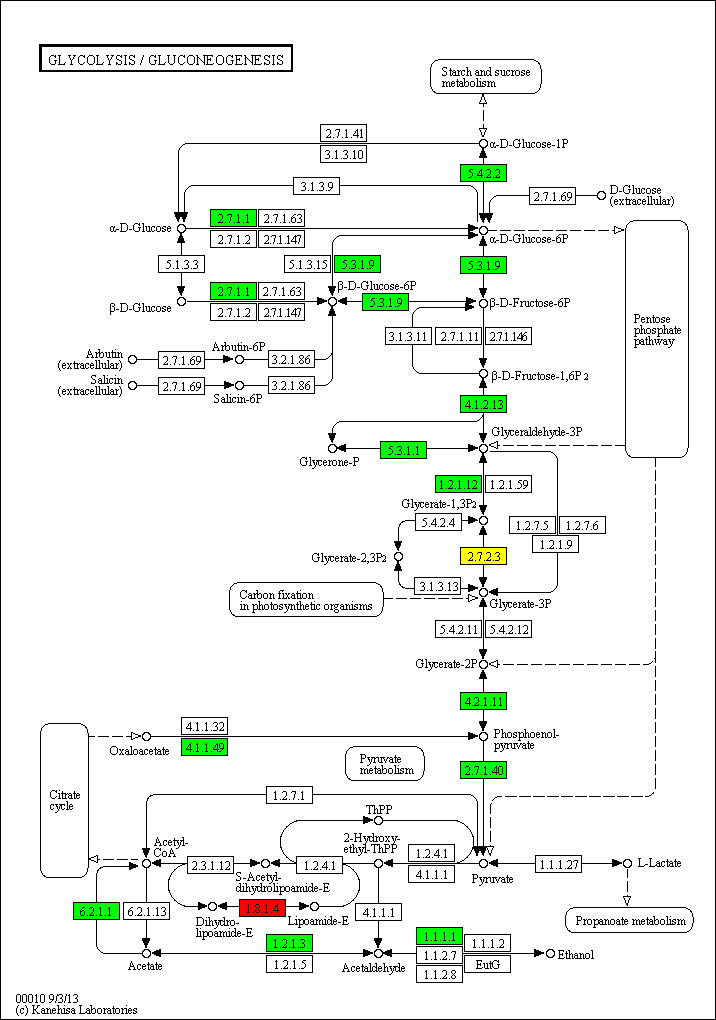

Supplement: Supplementary file 1 [file MBO3-6-na-s001.zip › mbo3485-sup-0005-FigS5.jpg]

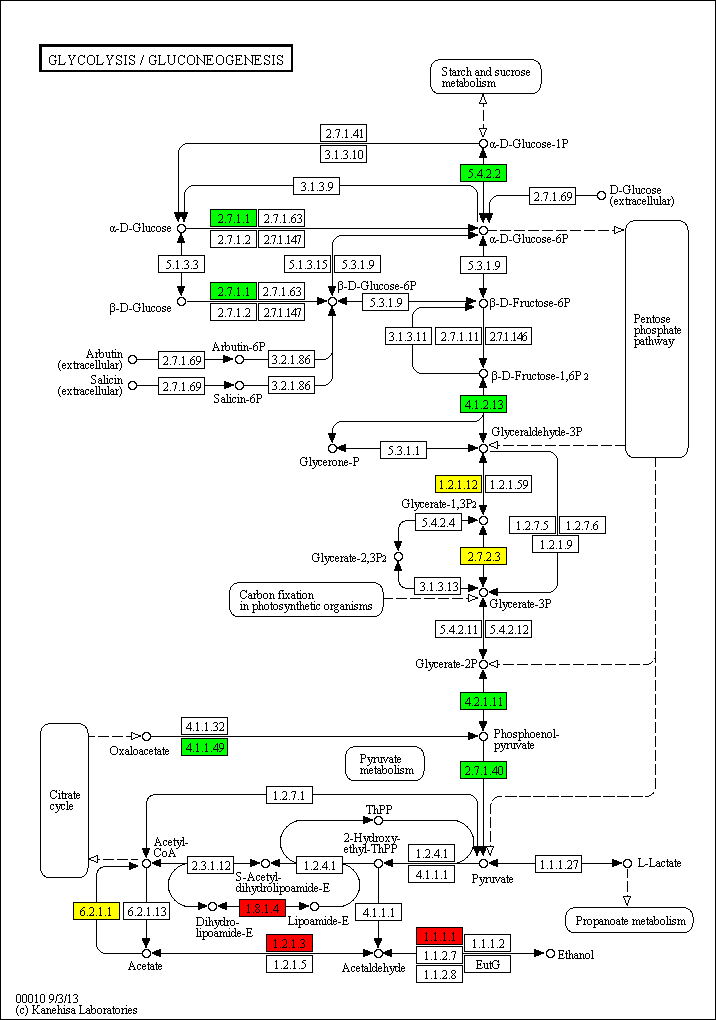

Supplement: Supplementary file 1 [file MBO3-6-na-s001.zip › mbo3485-sup-0006-FigS6.jpg]

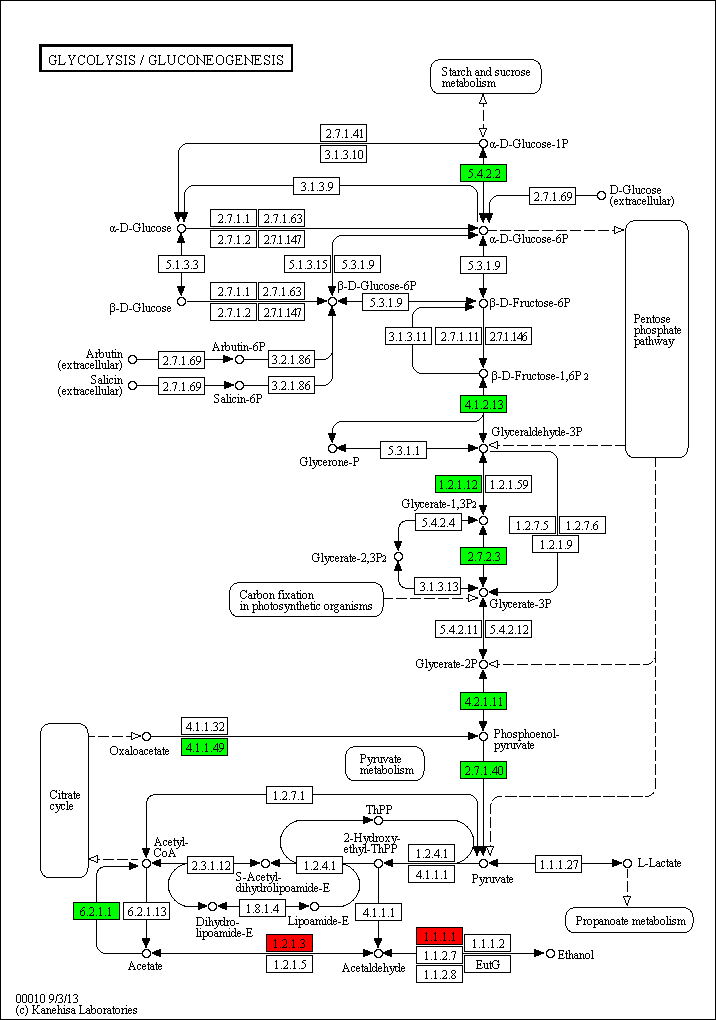

Supplement: Supplementary file 1 [file MBO3-6-na-s001.zip › mbo3485-sup-0007-FigS7.jpg]

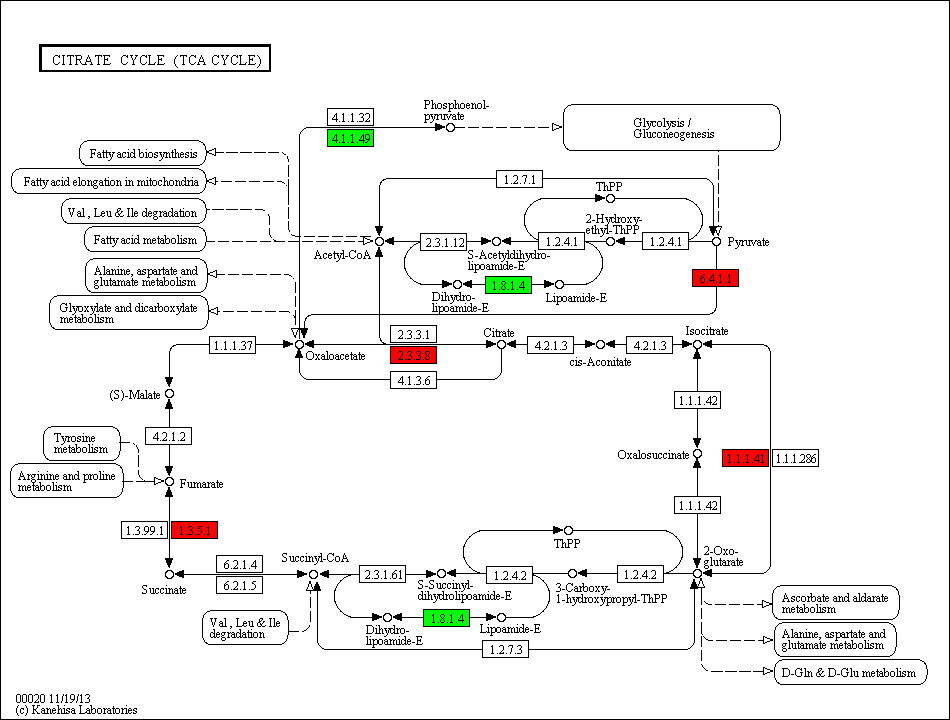

Supplement: Supplementary file 1 [file MBO3-6-na-s001.zip › mbo3485-sup-0008-FigS8.jpg]

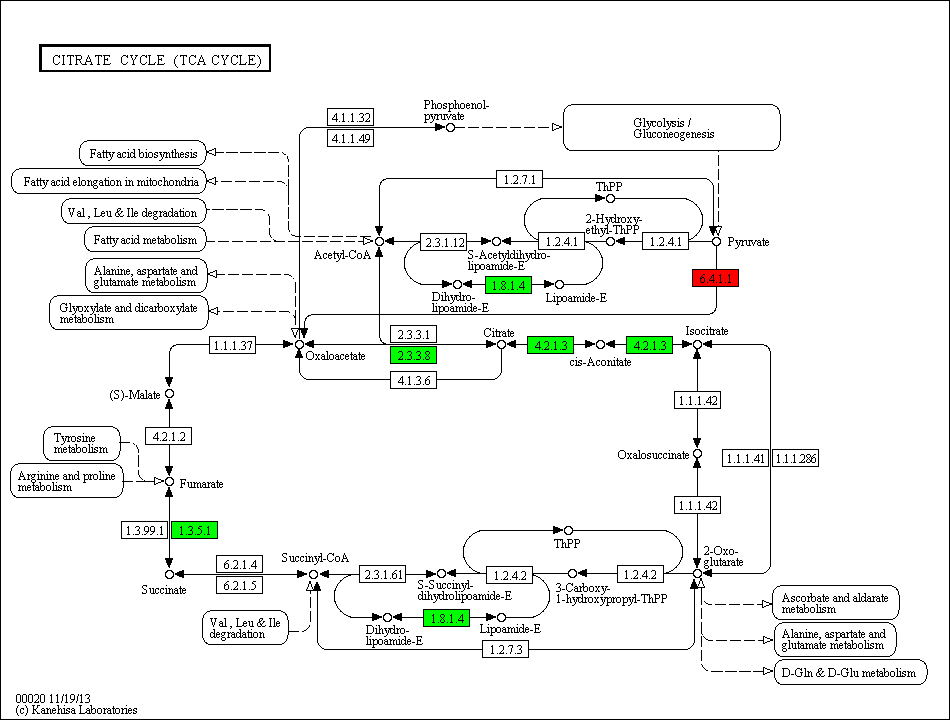

Supplement: Supplementary file 1 [file MBO3-6-na-s001.zip › mbo3485-sup-0009-FigS9.jpg]

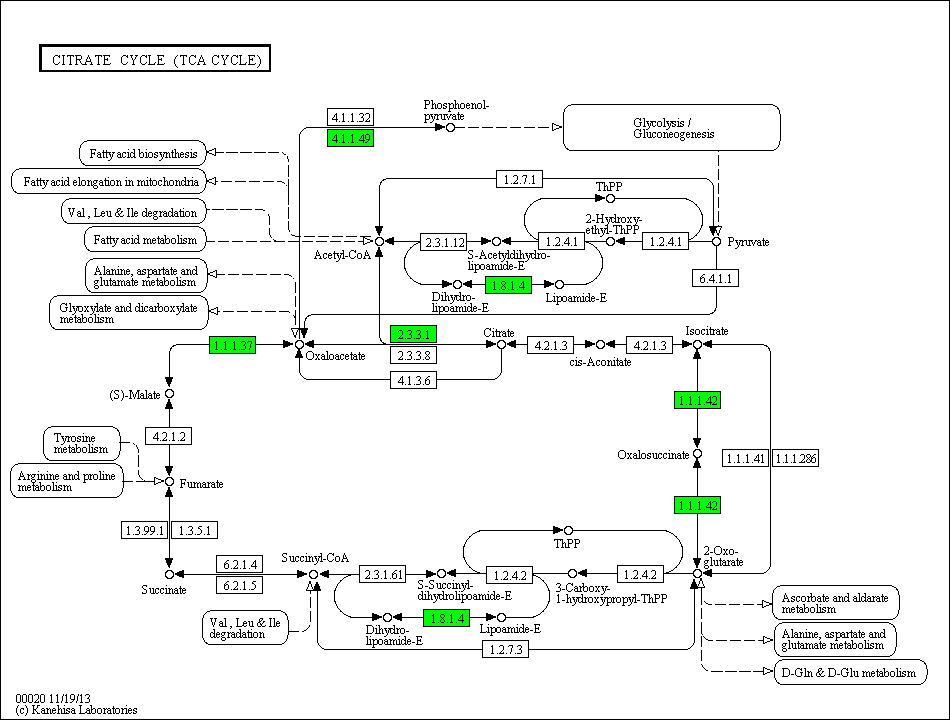

Supplement: Supplementary file 1 [file MBO3-6-na-s001.zip › mbo3485-sup-0010-FigS10.jpg]

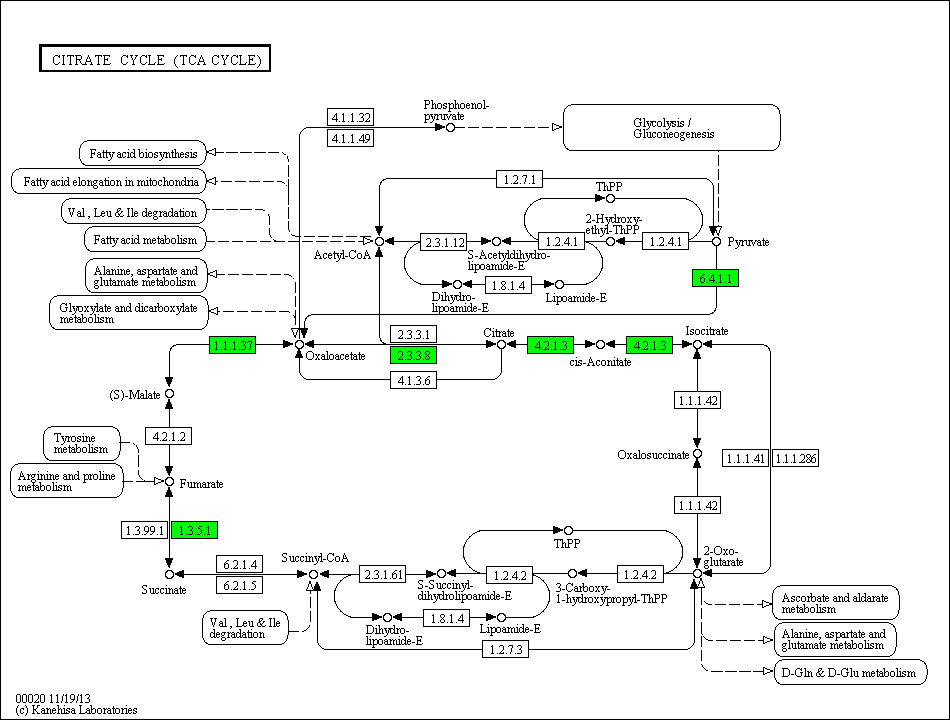

Supplement: Supplementary file 1 [file MBO3-6-na-s001.zip › mbo3485-sup-0011-FigS11.jpg]

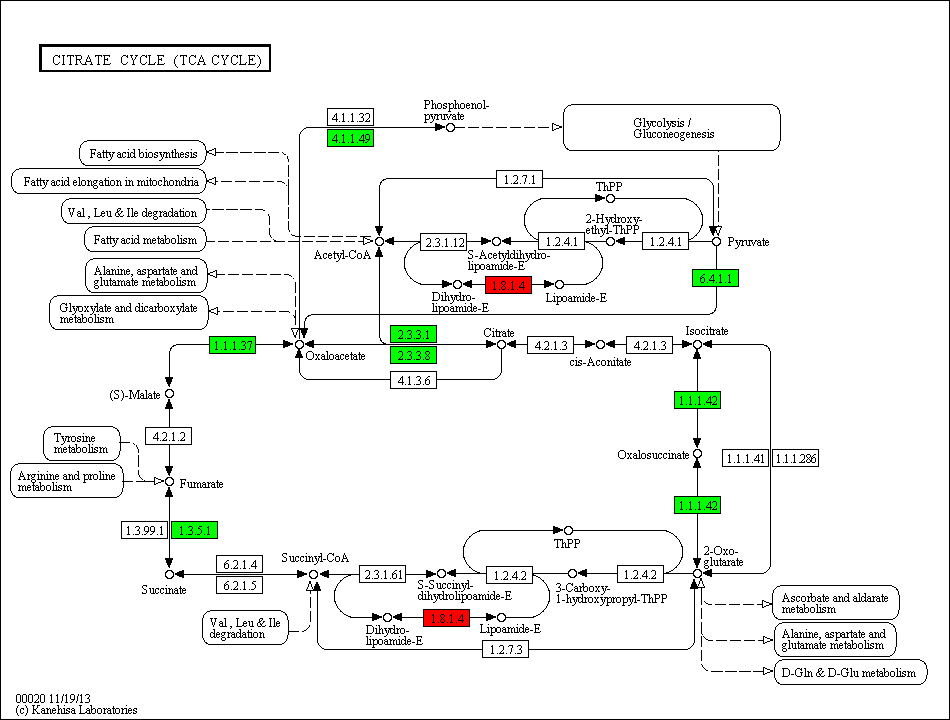

Supplement: Supplementary file 1 [file MBO3-6-na-s001.zip › mbo3485-sup-0012-FigS12.jpg]

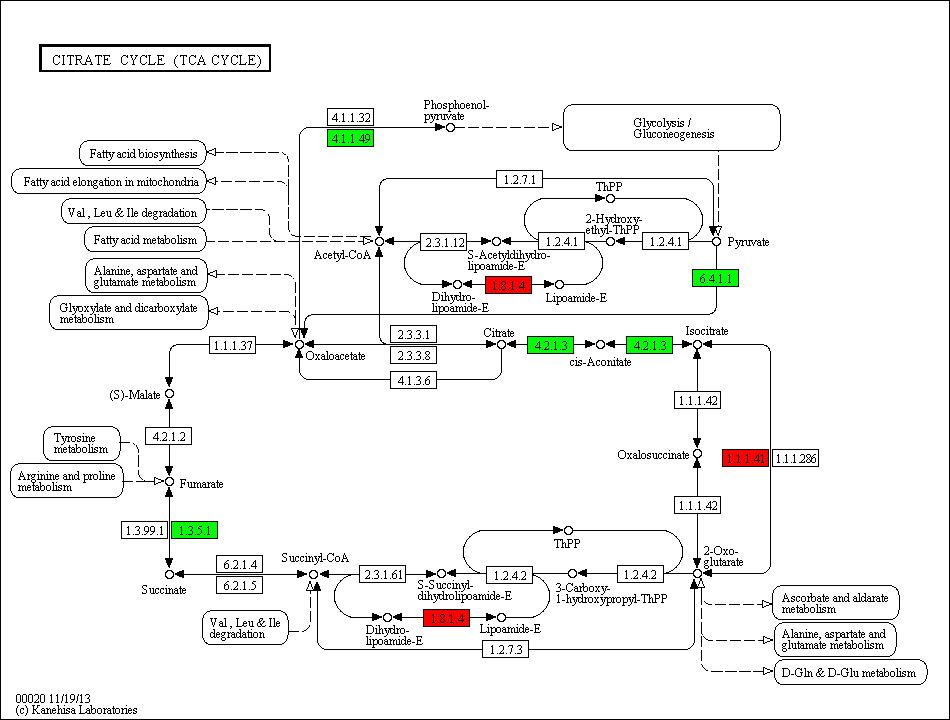

Supplement: Supplementary file 1 [file MBO3-6-na-s001.zip › mbo3485-sup-0013-FigS13.jpg]

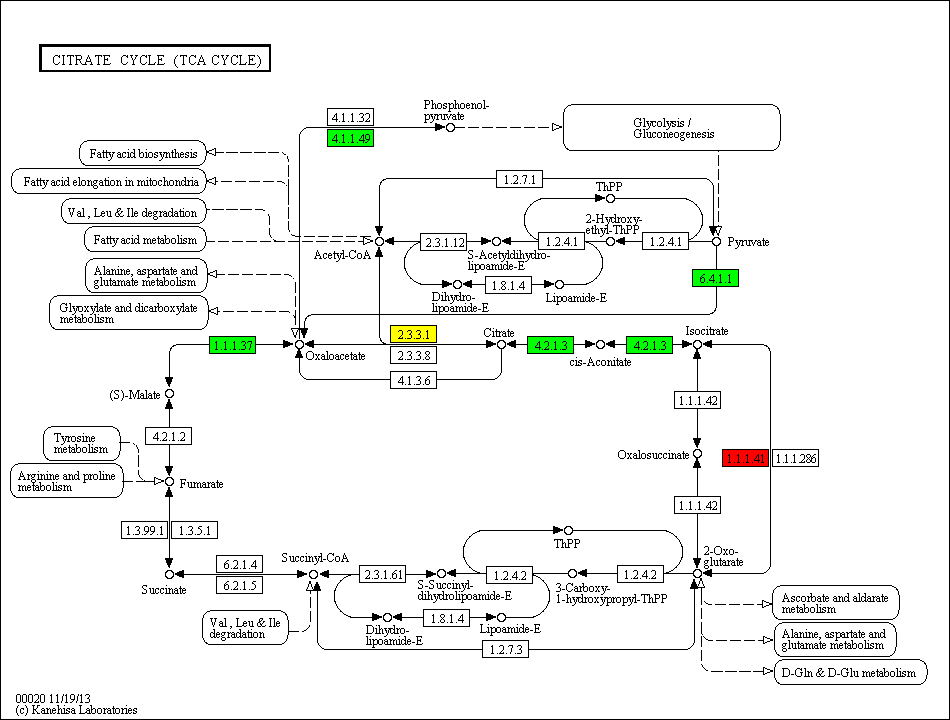

Supplement: Supplementary file 1 [file MBO3-6-na-s001.zip › mbo3485-sup-0014-FigS14.jpg]

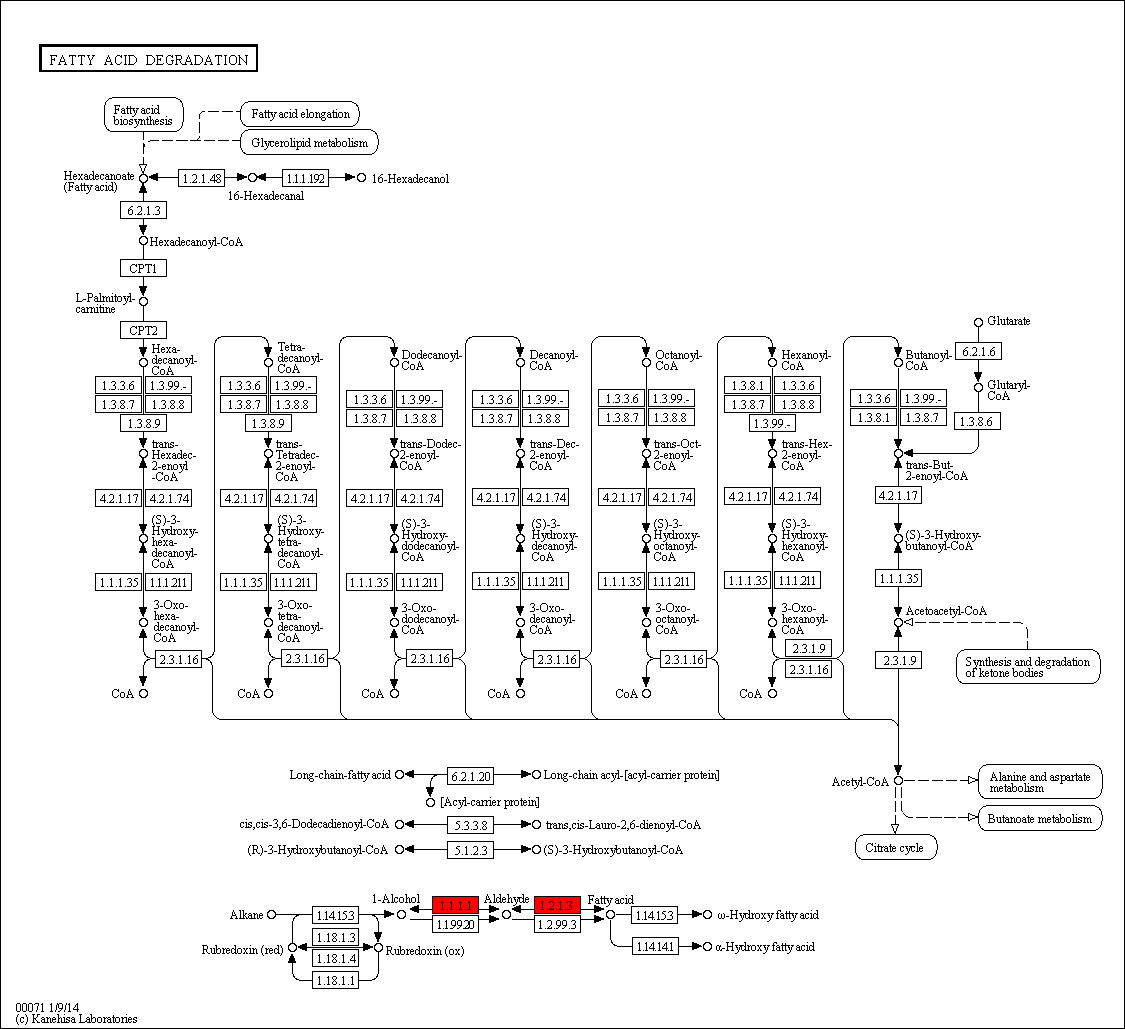

Supplement: Supplementary file 1 [file MBO3-6-na-s001.zip › mbo3485-sup-0015-FigS15.jpg]

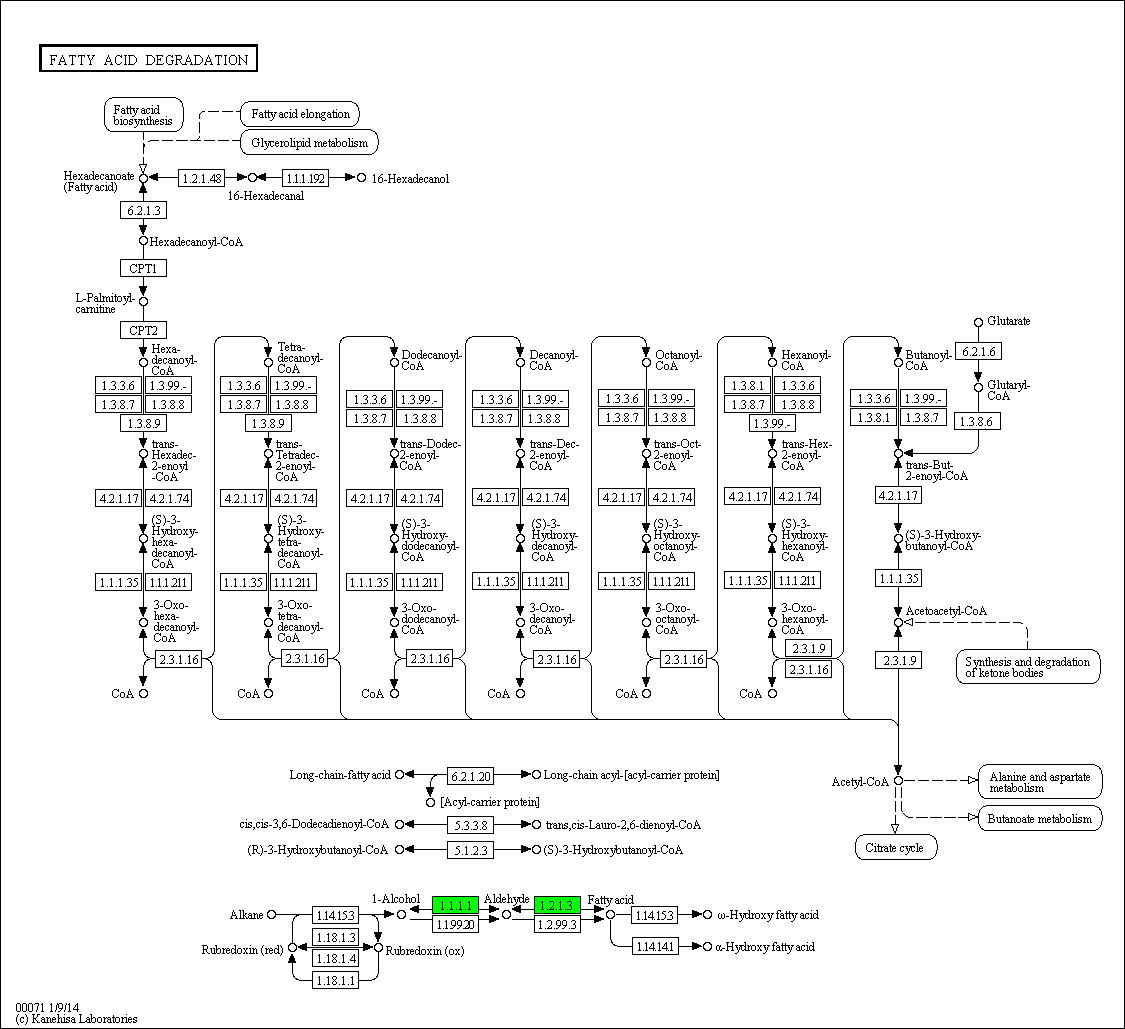

Supplement: Supplementary file 1 [file MBO3-6-na-s001.zip › mbo3485-sup-0016-FigS16.jpg]
