# Supplementary material for: A proteomic‐based investigation of potential copper‐responsive biomarkers: Proteins, conceptual networks, and metabolic pathways featuring Penicillium janthinellum from a heavy metal‐polluted ecological niche
Source: Microbiologyopen. 2017 May 9;6(4):e00485. doi: 10.1002/mbo3.485 (PMC5552966; doi:10.1002/mbo3.485)
Supplement: Supplementary file 1 [file MBO3-6-na-s001.zip › mbo3485-sup-0023-TableS2.doc]

**Table S2** The operating parameters of Proteome Discoverer 1.3 software for quantitative analysis of mass spectra

| **Name** | **Parameter option** |
| --- | --- |
| Protein ratio Type | Median |
| Minimum peptides | 1 |
| Normalisation method | Median |
| P-value | < 0.05 |
| Differential fold threshold | >1.2 |
