# Supplementary material for: A proteomic‐based investigation of potential copper‐responsive biomarkers: Proteins, conceptual networks, and metabolic pathways featuring Penicillium janthinellum from a heavy metal‐polluted ecological niche
Source: Microbiologyopen. 2017 May 9;6(4):e00485. doi: 10.1002/mbo3.485 (PMC5552966; doi:10.1002/mbo3.485)
Supplement: Supplementary file 1 [file MBO3-6-na-s001.zip › mbo3485-sup-0025-TableS4.doc]

| **Table S4** High-abundance proteins and some proteins **of** interest from the identified proteins | |
| --- | --- |
| **Protein** | **Number** |
| Hsp | 21 |
| 60S ribosomal protein | 17 |
| ATP-dependent RNA helicase | 17 |
| 40S ribosomal protein | 16 |
| Eukaryotic translation initiation factor | 16 |
| Actin | 13 |
| Elongation factor 1 | 11 |
| Histone | 10 |
| 26S protease regulatory subunit | 10 |
| Proteasome | 9 |
| Protein transport protein | 9 |
| ATP synthase subunit | 8 |
| Glyceraldehyde-3-phosphate dehydrogenase | 8 |
| Vacuolar protease/protein | 7 |
| GTP-binding protein | 6 |
| Catalase and catalase-peroxidase | 6 |
| NADH-cytochrome b5 | 5 |
| Phosphoglycerate kinase | 5 |
| SOD | 5 |
| Tubulin | 5 |
| NADH-ubiquinone oxidoreductase | 4 |
| V-type proton ATPase | 4 |
| RuvB-like helicase | 4 |
| 14-3-3 protein homolog | 2 |
| Spermidine synthase | 1 |
